# Occurrence and expression of genes encoding methyl-compound production in rumen bacteria

**DOI:** 10.1186/s42523-019-0016-0

**Published:** 2019-11-14

**Authors:** William J. Kelly, Sinead C. Leahy, Janine Kamke, Priya Soni, Satoshi Koike, Roderick Mackie, Rekha Seshadri, Gregory M. Cook, Sergio E. Morales, Chris Greening, Graeme T. Attwood

**Affiliations:** 1 Donvis Ltd, Palmerston North, New Zealand; 2 AgResearch Ltd, Grasslands Research Centre, Palmerston North, New Zealand; 3 Horizons Regional Council, Palmerston North, New Zealand; 4 Hokkaido University, Sapporo, Japan; 5 University of Illinois, Urbana, IL USA; 6 Department of Energy, Joint Genome Institute, San Francisco, CA USA; 7 University of Otago, Dunedin, New Zealand; 8 Monash University, Melbourne, Australia

**Keywords:** Rumen, Bacterial, Methyl-compound, Methanol, Methylamines

## Abstract

**Background:**

Digestive processes in the rumen lead to the release of methyl-compounds, mainly methanol and methylamines, which are used by methyltrophic methanogens to form methane, an important agricultural greenhouse gas. Methylamines are produced from plant phosphatidylcholine degradation, by choline trimethylamine lyase, while methanol comes from demethoxylation of dietary pectins via pectin methylesterase activity. We have screened rumen metagenomic and metatranscriptomic datasets, metagenome assembled genomes, and the Hungate1000 genomes to identify organisms capable of producing methyl-compounds. We also describe the enrichment of pectin-degrading and methane-forming microbes from sheep rumen contents and the analysis of their genomes via metagenomic assembly.

**Results:**

Screens of metagenomic data using the protein domains of choline trimethylamine lyase (CutC), and activator protein (CutD) found good matches only to *Olsenella umbonata* and to *Caecibacter*, while the Hungate1000 genomes and metagenome assembled genomes from the cattle rumen found bacteria within the phyla Actinobacteria, Firmicutes and Proteobacteria. The *cutC* and *cutD* genes clustered with genes that encode structural components of bacterial microcompartment proteins. *Prevotella* was the dominant genus encoding pectin methyl esterases, with smaller numbers of sequences identified from other fibre-degrading rumen bacteria. Some large pectin methyl esterases (> 2100 aa) were found to be encoded in *Butyrivibrio* genomes. The pectin-utilising, methane-producing consortium was composed of (i) a putative pectin-degrading bacterium (phylum Tenericutes, class *Mollicutes*), (ii) a galacturonate-using *Sphaerochaeta* sp. predicted to produce acetate, lactate, and ethanol, and (iii) a methylotrophic methanogen, *Methanosphaera* sp., with the ability to form methane via a primary ethanol-dependent, hydrogen-independent, methanogenesis pathway.

**Conclusions:**

The main bacteria that produce methyl-compounds have been identified in ruminants. Their enzymatic activities can now be targeted with the aim of finding ways to reduce the supply of methyl-compound substrates to methanogens, and thereby limit methylotrophic methanogenesis in the rumen.

## Background

Methane (CH_4_) is an important greenhouse gas (GHG) accounting for ~ 14% of total global GHG emissions [1]. Approximately 40% of this comes from agriculture, with the single largest source being enteric fermentation in ruminants. Ruminants are important to the economies of many developed and developing countries, and finding ways to reduce CH_4_ emissions from ruminants is a challenge facing farmers worldwide [2]. As a consequence of the digestive processes in the rumen, by-products of fibre degradation and fermentation end-products, including hydrogen (H_2_), carbon dioxide (CO_2_), methanol, methylamines, and methylsulphides, are formed but not used by the host animal. Hydrogenotrophic and methylotrophic methanogens in the rumen are able to remove these end products by reducing them to CH_4_, which is eructated from the animal leading to atmospheric emissions of CH_4_ [3]. Hydrogenotrophic rumen methanogens mainly belong to the genus *Methanobrevibacter*, while the core rumen methylotrophic methanogens are from the genus *Methanosphaera* and the order *Methanomassiliicoccales* [3].

The main methyl-compounds found in the rumen are methanol and methylamines. Methanol is present from around 0.8 mM in the rumen of cattle fed hay and grain [4] to around 0.07 mM in Brahman steers fed Rhodes grass hay [5] and is thought to be derived from demethyoxylation of dietary pectins via the action of pectin methyl esterases (PMEs; EC3.1.1.11). Pectin is a significant component of the plant cell wall (PCW) after cellulose, hemicellulose and lignin, and is found in the middle lamellae that joins cells together. While research with environmental bacteria has stressed the importance of pectin degradation in the initiation of PCW breakdown [6], little is known about the organisms that carry out pectin degradation and methanol release in the rumen. The rumen bacterium *Lachnospira multipara* produces pectin lyase (PL) and PME activities [7, 8] and has been regarded as the primary pectin fermenter isolated from rumen contents of animals fed diets high in pectin [9]. During pectin fermentation by *L. multipara*, methanol is formed as a product of PME activity [10], and pectin fermentation can cross-feed methanol-utilising bacteria such as *Eubacterium limosum* as has been demonstrated with co-cultures of these species [11]. However, *Lachnospira* is not normally abundant in the rumen [12], and other more abundant genera with pectin-degrading ability, notably *Butyrivibrio* and *Prevotella,* are likely to be the major pectin degraders.

Mono-, di- and tri-methylamines are produced mainly as the end-product of plant phosphatidylcholine degradation [13] via choline. Methylamine has been measured at around 0.085 mM in the rumen fluid of dairy cows fed a cereal grain diet [14] and ranges from 0.0285 to 0.703 mM in the rumen of cows fed varying amounts of barley grain [15] and from 0.334 to 0.564 mM in Brahman steers on the tropical forage, Rhodes grass [5]. Very little is known about how methylamines are produced in the rumen. It has been shown that labelled choline dosed into the rumen was rapidly metabolized to trimethylamine (TMA) by rumen microorganisms and the labelled methyl groups ended up as CH_4_ [16, 17]. A more recent study found a negative relationship between rumen Methanomassiliicoccales populations and urinary trimethylamine-*N*-oxide (TMAO) concentration [18], thought to be due to Methanomassiliicoccales using TMA for methane formation in the rumen, and diverting it from being oxidised to TMAO in the liver. More is known about the metabolism of choline and TMA in the human gut, as the TMAO formed in the liver is correlated with atherosclerosis in animal models and is linked with cardiovascular risks in human clinical trials [19, 20]. The release of TMA from choline was reported in the human gut bacterium, *Proteus mirabilis*, mediated by the enzyme choline trimethylamine lyase (CTMAL; EC:4.3.99.4) [21]. Microbial choline TMA lyase was found to be an enzyme complex composed of a catalytic choline utilisation polypeptide CutC, and an associated activating protein CutD, encoded by adjacent genes within a gene cluster that also contains genes encoding bacterial microcompartment proteins [22]. This gene cluster was first described from the rumen sulphate reducing bacterium, *Desulfovibrio desulfuricans*, and confining this activity within a bacterial microcompartment is seen as a means to prevent the volatile and toxic acetaldehyde intermediate damaging other cellular processes [22]. Several other human gut bacteria with choline TMA lyase activity have been identified [23, 24] and gut metagenomes have been screened for TMA-producing catabolic genes [25].

In order to target ruminal CH_3_-compound formation as a means to reduce methanogenesis, the types of organisms producing CH_3_-compounds in the rumen and the enzymes involved need to be identified. Here, we report a survey of rumen-derived metagenomic and metatranscriptomic datasets [26] and rumen metagenome assembled genomes [27] to identify the genes encoding the production of CH_3_-compounds, and which organisms express these genes under conditions prevailing in the rumen. We also screen the Hungate1000 genomes [28] for the occurrences of these genes and examine their arrangement within each genomic context, to give additional insights into the potential physiological context and genetic regulation of processes leading to CH_3_-compound release. Furthermore, we describe an enrichment culture experiment using pectin to encourage the growth of methanol-forming microbes from sheep rumen contents, and report the identification and analysis of metagenome assembled genomes (MAGs) from this enrichment.

## Results

### Identification of genes encoding production of mono-, di- and tri-methylamines

**Fig. 1:**
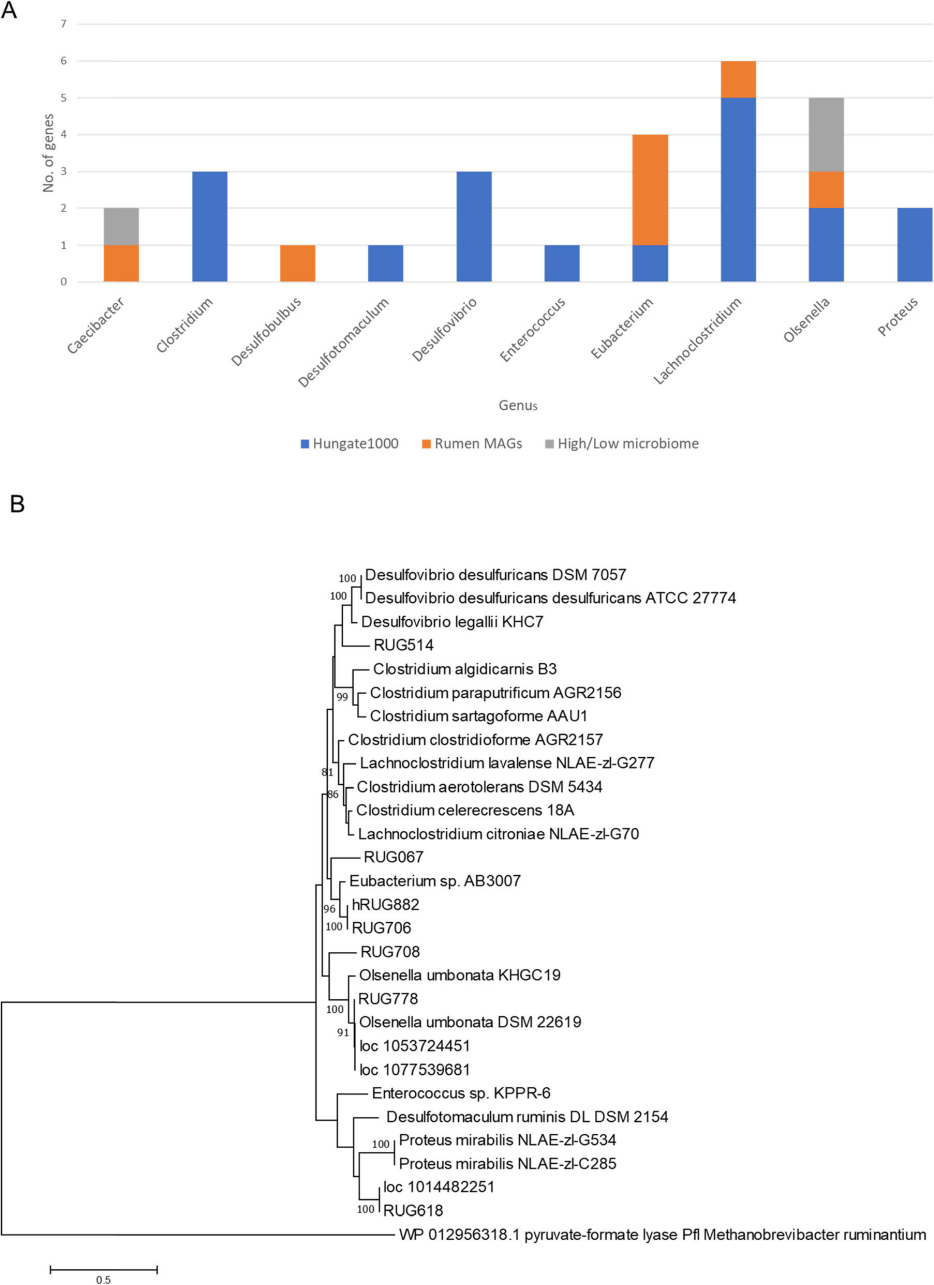
Choline TMA lyase (*cutC*) gene abundance and diversity in a combined rumen metagenome and metatranscriptome dataset, metagenome-assembled genomes and Hungate1000 bacterial genomes (**a**), and a phylogenetic tree showing the relationships of CutC protein sequences from all these sources (**b**)

The presence of genes encoding choline TMA lyase and the associated choline TMA lyase activator in rumen metagenome datasets was determined using the HMM models for CutC and CutD [25]. Analyses against the combined assembly of metagenome and metatranscriptome reads derived from rumen contents of sheep selected for differences in CH_4_ yield (11,801,660 ORFs) [26] revealed good matches for both CutC and CutD from *Olsenella umbonata* (Actinobacteria, *Coriobacteriaceae*, two hits) and *Caecibacter* (Firmicutes, *Veillonellaceae*, one hit), but to no other organisms. (Figure 1a, Additional file 1: Table S1A = CutC MG&MT sheet). CutC transcript abundances were low in the sheep metatranscriptome dataset, suggesting low level expression of these genes in the rumen of these animals. The contigs were quite short in the combined assembly so it was not possible to get an indication of the genome context from these data. However, examination of the SPADES re-assembled metagenomes from the same study has provided additional information on the genome context for these genes (Additional file 2: Figure S1A). Analysis against the predicted ORFs of 913 cattle rumen MAGs) [27] indicated that just seven MAGs contained a putative CutC gene (Fig. 1a).

The Hungate1000 Collection genomes were also screened for CutC and/or CutD domains (Table 1) and a phylogenetic tree of CutC sequences retrieved from rumen genome and metagenome/metatranscriptome sources is shown in Fig. 1b. In all cases the CutC and CutD genes were part of a larger cluster which included genes for the structural components of bacterial microcompartment proteins (Additional file 2: Figure S1B&C). A total of 18 bacterial strains were identified, 10 of rumen origin and 8 from faeces. None of these bacterial genera are regarded as abundant or prevalent members of the rumen microbiome based on results from the Global Rumen Census study [12]. The abundance of CutC sequences identified from Hungate1000 Collection genomes were assessed in the high and low methane yield sheep metagenome and metatranscriptome datasets (Additional file 1: Table S1A = CutC MG&MT table). The CutC from *Olsenella umbonata* DSM 22619 was most abundant in the metagenome dataset, followed by *Eubacterium* sp. AB3007 and *Desulfovibrio legallii* KHC7. CutC transcripts from Hungate1000 Collection genomes were mainly from *D. desulfuricans* subsp. *desulfuricans* ATCC 27774, *D. legallii* KHC7 and *O. umbonata* DSM 22619. Genes encoding CutC also include two non-specific Pfam domains (Pfam01228: glycine radical and Pfam02901: pyruvate formate lyase-like), but a further search using these domains did not find additional examples of choline TMA lyase.

**Table 1:** Choline TMA lyase gene occurrence in rumen microbial datasets

Rumen bacteria	Phylum	Strain	IMG gene ID	Locus Tag	aa	Origin
Hungate1000 [28]						
Rumen bacteria						
*Olsenella umbonata*	Actinobacteria	A2 (DSM 22619)	2608576534	Ga0059087_11836	847	sheep
*Olsenella umbonata*	Actinobacteria	KHGC19	2663665377	Ga0104367_1620	847	cow
*[Eubacterium]* sp*.*	Firmicutes	AB3007	2561437736	P156DRAFT_0760	847	cow
*Enterococcus* sp*.*	Firmicutes	KPPR-6	2608578278	Ga0059091_105198	857	cow
*Lachnoclostridium aerotolerans*	Firmicutes	X8A62 (DSM 5434)	2558962511	T546DRAFT_01546	848	sheep
*Clostridium sartagoforme*	Firmicutes	AAU1	2543441078	A500_11264	846	buffalo
*Desulfotomaculum ruminis*	Firmicutes	DL (DSM 2154)	650891141	Desru_2090	847	sheep
*Desulfovibrio legallii*	Proteobacteria	KHC7	2654384186	Ga0104380_103103	848	cow
*Desulfovibrio desulfuricans*	Proteobacteria	G11 (DSM 7057)	2595153453	IE73DRAFT_02008	848	cow
*Desulfovibrio desulfuricans subsp. desulfuricans*	Proteobacteria	Mb (ATCC 27774)	643582164	Ddes_1357	848	sheep
Faecal bacteria						
*Clostridium algidicarnis*	Firmicutes	B3	2562235685	BV55DRAFT_1892	847	cow
*Clostridium paraputrificum*	Firmicutes	AGR2156	2525457652	G594DRAFT_02041	846	calf
*Lachnoclostridium citroniae*	Firmicutes	NLAE-zl-G70	2624603054	Ga0070263_11089	855	goat
*Lachnoclostridium clostridioforme*	Firmicutes	AGR2157	2525451452	G614DRAFT_00825	853	calf
*Lachnoclostridium lavalense*	Firmicutes	NLAE-zl-G277	2624766256	Ga0070262_11929	846	goat
*Lachnoclostridium celerecrescens*	Firmicutes	18A (DSM 5628)	2596304492	H171DRAFT_4337	847	cow
*Proteus mirabilis*	Proteobacteria	NLAE-zl-G534	2624727893	Ga0066901_10954	1142	goat
*Proteus mirabilis*	Proteobacteria	NLAE-zl-C285	2656994829	Ga0104388_10857	1142	cow
Rumen metagenome assembled genomes (MAGs [27])						
hRUG882	Firmicutes	N/A	N/A	NODE_2309_length_24057 _cov_25.2708_2	857	cow
RUG067	Firmicutes	N/A	N/A	k87_66874274_20	872	cow
RUG514	Proteobacteria	N/A	N/A	k87_1124619_4	811	cow
RUG618	Firmicutes	N/A	N/A	k87_25912225_4	855	cow
RUG706	Firmicutes	N/A	N/A	scaffold_2105_5	857	cow
RUG708	Firmicutes	N/A	N/A	k87_13261599_27	860	cow
RUG778	Actinobacteria	N/A	N/A	scaffold_14892_2	848	cow
High/Low microbiome [26]						
NZ sheep rumen microbiome combined assembly	Actinobacteria	N/A	37167	loc_1053724451	799	sheep
NZ sheep rumen microbiome combined assembly	Actinobacteria	N/A	37167	loc_1077539681	398	sheep
NZ sheep rumen microbiome combined assembly	Firmicutes	N/A	37167	loc_1014482251	394	sheep

### Identification of genes encoding production of methanol

**Fig. 2:**
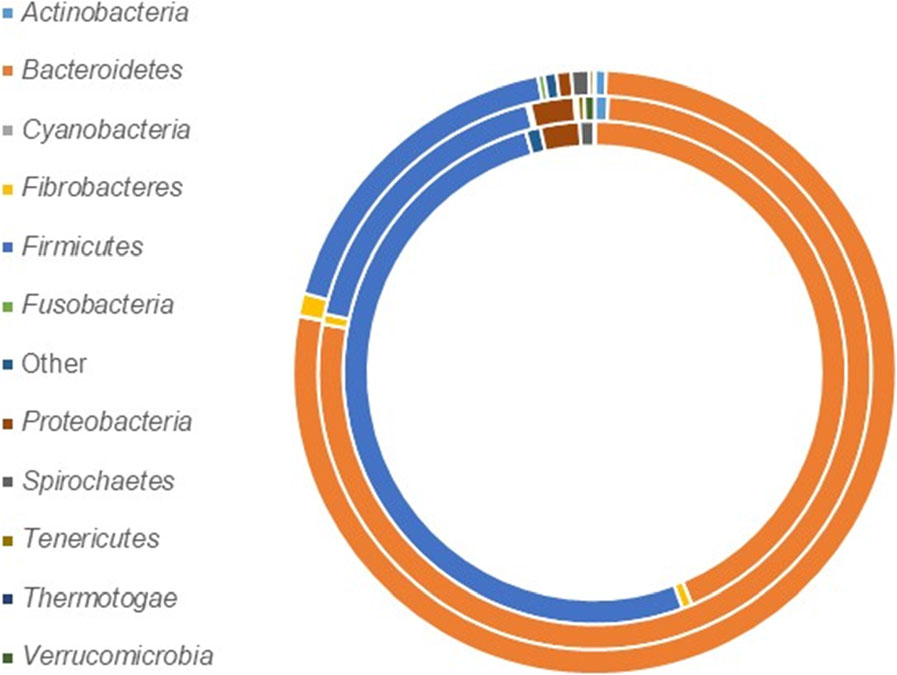
Abundance (%) and diversity of genes encoding pectin methyl esterase (PME; PF01095)-domain containing proteins in a combined rumen metagenome and metatranscriptome dataset (outer circle; *n* = 2414), metagenome-assembled genomes (middle circle; *n* = 505) and Hungate1000 bacterial genomes (inner circle; *n* = 315)

To determine the presence of genes for PMEs in rumen metagenome datasets, the HMM model for Pfam01095 (Pectinesterase) was used to search against the combined assembly of metagenome and metatranscriptome reads screened from rumen contents of sheep described above [26]. Using the HMM default settings, a total of 2414 hits were retrieved which were analysed using BLAST searches (Fig. 2; Additional file 1: Table S1B = PME MG sheet). The sequences of the top BLAST hits were almost entirely (2398) of bacterial origin. Of the bacterial sequences, 1012 (42%) gave a top BLAST hit to a rumen isolate from the Hungate 1000 Collection. *Prevotella* was the dominant genus with 475 sequences that gave top BLAST hits to rumen isolates, along with *Ruminococcus* (171), *Bacteroides* (147), *Butyrivibrio* (49), *Fibrobacter* (39), *Lachnospira* (19), *Oribacterium* (19), as well as unclassified *Lachnospiraceae* (19) and *Erysipelotrichaceae* (14). Only 63 of the 2414 BLAST hits (2.6%) were derived from ‘uncultured’ organisms. Of these, 61 matched to the same sequence (AEF12641) which encodes a 1501 aa protein, annotated as being from an uncultured *Prevotella* from a bovine rumen sample. This protein shows ~ 70% aa identity with PMEs from the rumen *Prevotella* strains TF2–5 and BPI-148. Many hits (115) show > 90% aa identity to PMEs from rumen bacterial isolates, the best matches (> 99% aa identity) were to *Prevotella bryantii* (4 different PMEs), *Lachnospira multipara* (3 different PMEs), *Ruminococcus* sp*.*, *Prevotella* sp., *Butyrivibrio* sp. and *Oribacterium* sp. The largest PMEs detected (> 2100 aa) were predominantly from *Butyrivibrio* spp. An analysis of PME transcript abundance also indicated that PMEs from *Prevotella* spp. were the most highly expressed (Additional file 1: Table S1C=PME MT sheet).

Similar results were obtained from a BLAST search analysis of the predicted ORFs from 913 cattle rumen MAGs [27]. This indicated the presence of 505 putative PME genes of bacterial origin (Additional file 1: Table S1D = PME RUG). Of these genes 146 (29%) gave a top BLAST hit to a rumen isolate from the Hungate 1000 Collection, with *Prevotella* again being the dominant genus. Only 5 ORFs derived from ‘uncultured’ organisms, and of these, 4 matched to the same sequence (AEF12641) that was observed in the combined assembly analysis. The largest PMEs detected were from *Butyrivibrio* spp.

Bacterial isolates from the human and pig gut microbiomes, and sequences from human microbiome metagenome studies, also made up significant numbers of the top BLAST hits retrieved from this analysis. Members of the genus *Prevotella* again provided the greatest number of sequences, and many of these sequences also give BLAST matches to rumen *Prevotella* isolates. This indicates, as previously reported [29], that more cultures are needed to capture the full diversity of rumen *Prevotella* species. Overall, 1394 sequences (58%) from the combined assembly and 245 ORFs (49%) from the MAGs gave a best match to sequences from members of the genus *Prevotella.* Further examination of these *Prevotella* results showed that 583 sequences (24%) from the combined assembly and 94 ORFs (19%) from the MAGs match to a PME of 324–330 aa, usually containing a signal peptide sequence at the N-terminus. *Prevotella* belongs to the phylum Bacteroidetes, and polysaccharide utilization is a characteristic feature within this group of organisms. The genes encoding polysaccharide breakdown are usually organised within polysaccharide utilization loci (PULs), which are defined as co-located genes organized around a *susCD* gene pair. PULs are thought to coordinate the breakdown of complex glycans via the carbohydrate-degrading enzymes co-located within the PUL. PULs are catalogued in the CAZy PUL database (PULDB) [30] which has recently been updated to include the Hungate1000 Collection genomes. Using the PULDB, the genomic context of the PME encoding genes was examined and most of the PME genes (including those encoding proteins of 324–330 aa) were found outside of PULs in the rumen *Prevotella,* even though they encode numerous examples of PULs in their genomes (ranging from 14 in *P. albensis*, up to 38 in *Prevotella* sp. strain KH1P2). However, several *P. bryantii* strains (B14, C21a, FB3001, KHPX14), *P. ruminicola* strains (D31d, Ga6B6, KHT3 AGR2160,) and *Prevotella* sp. strains (P6B1, P6B4, RM4, TC2–28, BPI-34, TF2–5) had one to three CE8 genes located within PULs. For example, in *P. bryantii* C21a two CE8 genes (G638DRAFT_00481, G638DRAFT_00861) were found in PULs 2 and 10 where they are co-located with genes for glycoside hydrolases and polysaccharide lyases suggesting that in this bacterium, pectin breakdown is a coordinated process.

The Hungate1000 Collection reference genome set was searched using information from the CAZy (http://www.cazy.org/) database (carbohydrate esterase family 8, CE8) and the protein domain specific for PME (Pfam01095), with the results shown in Additional file 3: Table S2. A total of 315 genes encoding PMEs were found in 159 microbial strains with up to six different PME-encoding genes found in a single strain. Strains belonging to the phylum Bacteroidetes showed the highest prevalence of PME genes. Many of the predicted PMEs contained signal peptide sequences, indicating a cell-surface or extracellular location. In addition, several genes encoded large multi-domain proteins, the most commonly associated domains included pectate lyases (Pfams 00544 and 09492), hydrolases (lipases/esterases Pfams 07859 and 13,472) and putative cell surface-binding components (Pfams 01473, 13,149 and 13,205).

### Pectin enrichment culture from sheep rumen contents

While the above analyses focused on individual organisms and highlighted the detection of their genes in metagenomic and metatranscriptomic rumen datasets, a complementary aspect of the current study was to investigate the inter-relationships between members of the rumen microbial community that provide methylotrophic substrates for methanogenesis. To achieve this we carried out an enrichment experiment using homogalacturonan pectin (methyloxylated polygalacturonic acid) as a potential source of methanol, which in turn would act as the substrate for methanogenesis. A pectin-utilising, methane-producing enrichment was established and DNA extracted from the resulting microbial consortium was sequenced (BioProject accession: PRJNA365034).

**Fig. 3:**
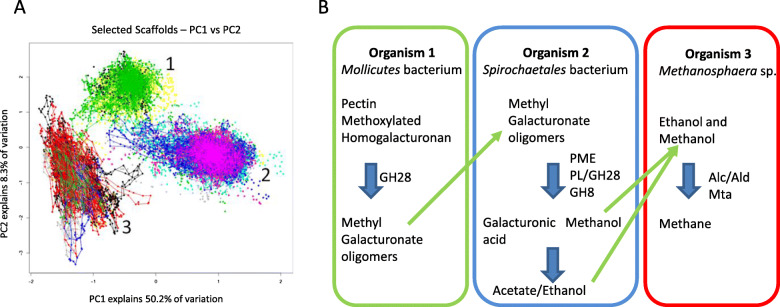
**a**: Tetranucleotide plot of the 24 scaffolds defining the 3 organisms recovered from a pectin-utilising, methane-producing enrichment culture. **b**: Predicted metabolic relationship between the three organisms enabling pectin conversion to methane

The consortium metagenome sequences assembled into 107 contigs and MetaBAT analysis grouped the 24 largest scaffolds into three bins, each of which represent uncultivated members of the rumen microbiome (Fig. 3a; Additional file 4: Table S3). The assembled genome of Organism 1 consisted of three contigs, with a combined size of 1.46 Mb and a GC content of ~ 38%. CheckM analysis indicated the assembled genome was 99.39% complete with 0% contamination. The 16S and 23 rRNA genes did not show a close relationship with any cultivated organisms, the closest matches being to members of the family Erysipelotrichaceae. Examination of the gene complement of Organism 1 identified a small number of genes encoding carbohydrate active enzymes (CAZymes), including members of glycoside hydrolase families GH10, GH32, GH43, GH53 and GH65, which indicates an ability to ferment plant polysaccharides. It also encodes genes for tandem signal peptide-containing polygalacturonases (GH28) which show weak homology (~ 40% nucleotide identity) to metagenome assembled genomes from environmental Tenericutes [31]. The second of these polygalacturonases contains a CBM32 domain (Pfam00754) which has been shown to mediate binding to polygalacturonate [32].

Organism 2 had 11 contigs associated with its assembled genome, giving a size of 3.61 Mb with a GC content of ~ 52%. CheckM analysis indicated 97.13% genome completeness with 0% contamination. The 16S rRNA gene found on one contig gives top BLAST hits to members of the genus *Sphaerochaeta* at ~ 91% identity, placing this organism in the phylum Spirochaetes. This organism appears to share the key features that distinguish *Sphaerochaeta* from most Spirochaetes, namely the lack of motility and non-spiral morphology; analysis of the genome indicated the absence of motility and chemotaxis genes, while examination of the enrichment culture by phase contrast microscopy did not show the presence of organisms with helical morphology characteristic of other members of the Spirochaetes phylum. The genome of Organism 2 also encodes numerous carbohydrate metabolism and fermentation genes [33], including a PME, a pectate lyase/polygalacturonase and six GH88 family unsaturated glucuronyl hydrolases predicted to mediate homogalacturonan metabolism. The PME, the pectate lyase/polygalacturonase and three of the GH88 proteins show homology (~ 62–84% aa identity) with a *Spirochaetales* MAG from activated sludge. None of the predicted proteins have signal peptide sequences indicating they function intracellularly. However, a large number of ABC carbohydrate transporters were identified, including 52 substrate-binding proteins identified as belonging to COG1653, which is frequently associated with the uptake of oligosaccharides. A pectinesterase gene with a best BLAST match to *Sphaerochaeta coccoides* DSM 17374 was also identified from one of the cattle rumen MAGs (RUG703).

**Fig. 4:**
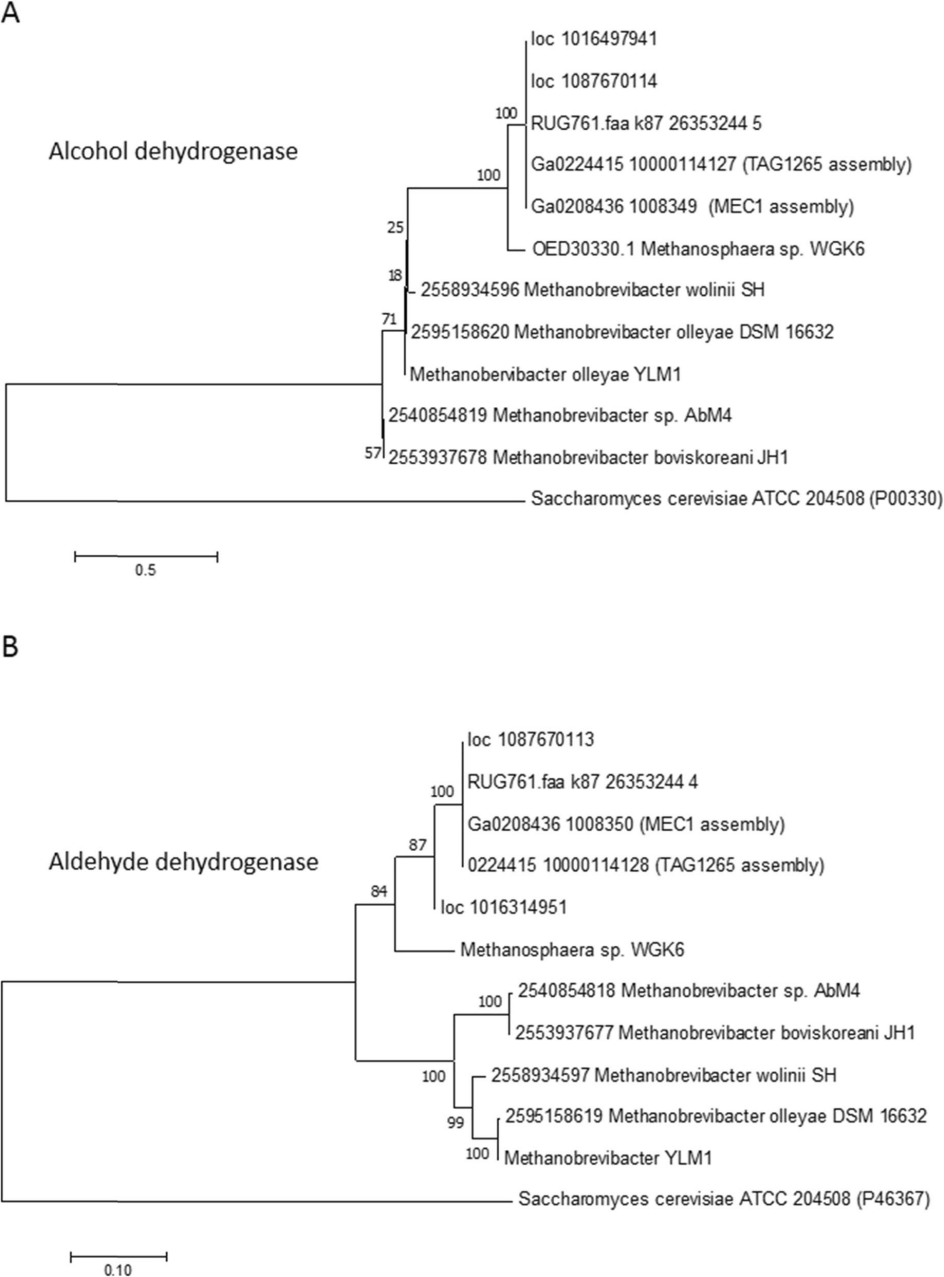
Phylogenetic analysis of alcohol dehydrogenase (**a**) and aldehyde dehydrogenase (**b**) genes from rumen methanogen genomes and rumen MAGs. Both trees were constructed with the Jones-Taylor Thornton (JTT) model. *Saccharomyces cerevisiae* ATCC 204508 was used as the outgroup. Numbers represent the relative frequency of branch clustering based on 1000 bootstrap runs, bootstrap values < 50% are removed. Rumen MAGs; MEC1, Organism 3 (*Methanosphaera* sp.) from the pectin enrichment culture in this study; TAG1265, metagenome assembled *Methanosphaera* sp. sequences from low methane yield sheep datasets [35]; RUG761, metagenome assembled *Methanosphaera* sp. sequences from cattle [27]

Ten contigs were associated with a third organism predicting a genome size of 2.0 Mb and a GC content of ~ 30%. CheckM analyses indicated that the assembled genome was 97.6% complete with 0% contamination. The 16S rRNA gene of Organism 3 gave a top hit to the type strain of *Methanosphaera stadtmanae* at 97% identity. Members of the genus *Methanosphaera* are methylotrophic methanogens [34], but although they are known to be present in the rumen from community profiling [3] only a few rumen isolates are available for study [35]. The assembled genome encodes the genes required for producing methane from methanol, but not from methylamines, and like *M. stadtmanae* DSM3091, lacks the genes for molybdopterin biosynthesis which suggests that it may be unable to reduce CO_2_ to methane due to the lack of this co-factor. Unlike *M. stadtmanae,* Organism 3 encodes a pair of genes encoding putative alcohol and aldehyde dehydrogenases which cluster with similar genes from *Methanosphaera* sp. WGK6 isolated from the wallaby gut [36], *Methanosphaera* sp. metagenome assembled genome from cattle (RUG761, [27]) and sheep (TAG1265, [35]), and more distantly with similar genes from the genome of the rumen methanogens, *Methanobrevibacter* sp. AbM4 [37] and *Mbb. boviskoreani* [38] (Fig. 4). Overall, the results from the analysis of the assembled genomes (Additional file 4: Table S3) show that these three organisms are likely to act together to convert pectin to methane (Fig. 3b).

## Discussion

Current rumen manipulation strategies targeting CH_4_ mitigation are focused on direct inhibition of methanogens, targeting their essential functions via small molecule inhibitors and antimicrobial peptides or surface proteins through methanogen-targeted vaccines [39]. There has been little exploration of the opportunities around manipulating the supply of substrates to methanogens. Methylotrophic methanogens in the rumen appear to be limited by the availability of CH_3_-compounds. The energy available from the reduction of methanol to CH_4_ (CH_3_OH + H_2_ → CH_4_ + H_2_O) is − 112.5 kJ/mole, compared to − 131 kJ/mole for the reduction of CO_2_ (CO_2_ + 4 H_2_ → CH_4_ + 2 H_2_O) [40] but reflecting reaction stoichiometries, methylotrophs require only 1 mole of H_2_ per mole of CH_4_, whereas hydrogenotrophs require 4 H_2_ per mole of CH_4_. This means that methylotrophs have a lower H_2_ threshold, and when the energy requirement for ATP biosynthesis is considered, methylotrophs always have a greater net free energy change than hydrogenotrophs under conditions prevailing in the rumen. However, despite this thermodynamic advantage, the hydrogenotrophic *Methanobrevibacter* spp. are the main methanogens making up 75–78% of the methanogenic archaea in the rumen, [3, 12]. This suggests that the growth of methylotrophic methanogens is governed by the availability of CH_3_-compounds rather than the dissolved H_2_ concentration. Nevertheless, methanogens capable of methylotrophic methanogenesis represent around 22–25% of the methanogens in the rumen and reducing their supply of CH_3_-compound substrates in the rumen offers an opportunity to target these methanogens to reduce CH_4_ formation.

Recent work on a global analysis of rumen microbial communities from ruminant species and microbiome characterisation studies [12, 26–28] have provided large datasets which can be used to identify the major rumen bacteria involved in releasing CH_3_-compounds from plant material, and the genes encoding these activities. Our screens for ruminal TMA production revealed surprisingly few genes and organisms involved in this process. A total of 18 bacterial strains were identified using the CutC/D HMM models, and they belong to the same three phyla (Actinobacteria, Firmicutes and Proteobacteria) that were identified in studies on TMA metabolism in the human gut [22, 25]. Overall it appears that TMA lyase and choline TMA lyase activator genes are rare in the rumen. None of the seven bacterial genera detected with these genes would be regarded as abundant or prevalent members of the rumen microbiome based on results from the Global Rumen Census study [12]. The metagenome/metatranscriptome dataset, indicate that *Olsenella* and *Caecibacter* are the main methylamine producers in sheep, while MAG-derived sequences indicate that organisms related to *Olsenella*, *Caecibacter*, and *Eubacterium* are likely to be important in cattle.

We used the pectinesterase Pfam (PF01095) (EC 3.1.1.11) to screen the rumen microbiome datasets for signatures of the methanol-producing enzyme, PME. Pectinesterase is commonly found in plants where it plays an important role in fruit ripening, but it is also found in plant pathogens where it is involved in the de-esterification of pectin into pectate and methanol during the breakdown of plant material. In the rumen, many organisms are involved in pectin degradation, and our screens identified the majority of the pectinesterase-containing organisms belonged to the genus *Prevotella*. The metagenome sequences were short (average of 253 aa) compared to the predicted full length of the PME proteins, which meant that it was not possible to get much genome context around these metagenomic and metatranscriptomic hits. The majority of the metagenome-derived PMEs were most similar to PMEs found in *Prevotella* genomes from the Hungate1000 Collection or reported from other gut environments. PME expression in *Prevotella* has been reported previously as part of a study investigating carbohydrate esterase activities involved in hemicellulose degradation [41]. The expression of *P. ruminicola* 23 pectin esterases, Pec E1 and Pec E2, were analysed during growth on differing carbohydrates; Pec E2 was found to be more than 2× upregulated on xylo-oligosaccharides derived from corn fiber relative to glucose, suggesting a potential role for this enzyme in hemicellulose degradation.

From our preliminary analysis it appears that *Prevotella* are the main providers of methanol in the rumen since they make up the bulk of the PME sequences. The particular prevalence of *Prevotella* PMEs in the 324–330 aa size range suggests that these enzymatic activities are significant contributors. From genomic analyses, it is likely that *Prevotella bryantii, Bacteroides* sp. KHT7*,* and *Lachnospira multipara* are specialised pectin degraders, while *Prevotella ruminicola* and other *Prevotella, Butyrivibrio,* and *Oribacterium* species are generalist bacteria with the ability to degrade pectin. Interestingly, the celluloytic bacteria *Fibrobacter succinogenes* and *Ruminococcus* spp. encode PMEs but are not able to use pectin for growth, and may therefore be using these activities for clearing away pectins to allow access to their primary substrate, cellulose.

The results of the pectin enrichment experiment add another dimension to this study, and showed the potential importance of members of the rumen microbiota distinct from those highlighted by analysis of individual genomes and metagenomes. Three genomes were assembled from the metagenome sequence of the pectin-enriched consortium and the analysis shows that the three organisms encoding these genomes likely act together to convert pectin to methane (Fig. 1). The 16S rRNA gene of Organism 1 was not closely associated with any cultivated organism, but the absence of genes involved in peptidoglycan biosynthesis in its genome, coupled with the predicted small genome size, strongly suggest that this organism is a member of the class *Mollicutes* in the phylum Tenericutes. There have been few studies of the rumen members of this bacterial group but they are characterised as having a fermentative metabolism and occurring in association with other rumen inhabitants [42]. The presence of CAZYmes GH10, GH32, GH43, GH53 and GH65, indicates a general ability to breakdown plant polysaccharides, while the presence of extracellular GH28 polygalacturonases with CBM32 polygalacturonate binding domains suggest some degree of pectin degradation ability. However, Organism 1 is probably unable to utilize the major products of homogalacturonan degradation as it does not encode a pectin methyl esterase or any of the enzymes from the galacturonate utilization pathway. Like the polysaccharide-degrading activities of other rumen bacteria [43, 44], Organism 1 may use its pectin-degrading activity to clear away pectin from plant cell walls and allow access to its preferred substrate, probably hemicelluloses.

In contrast, Organism 2 (*Sphaerochaeta* sp.) has the complete complement of genes encoding the enzymes necessary for galacturonate utilization, although it does not encode extracellular enzymes involved in this process. It has a well-developed uptake system for the products of pectin degradation, and likely transports the pectin degradation products of Organisms 1 to act as substrates for its growth. The PME encoded by this *Sphaerochaeta* sp. may act upon methoxylated oligogalacturonides to release methanol as a prelude to further depolymerisation and fermentation. The metabolic profile of the *Sphaerochaeta* sp. indicates that acetate, lactate, and ethanol would be also be formed from fermentation of pectin-derived substrates. These compounds are potential energy and carbon sources for Organism 3, the methylotrophic methanogen *Methanosphaera* sp., which has the gene complement required for producing methane from methanol. Furthermore, this *Methanosphaera* sp. has genes encoding putative alcohol and aldehyde dehydrogenases; in other methanogens these genes have been shown to allow ethanol to be used as a source of reducing power for methane production and growth in *Methanosphaera* sp. WGK6 [36], *Methanobrevibacter* sp. AbM4 [37, 45] and *Mbb. ruminantium* [39]. The strong similarities among these genes lead us to predict that the *Methanosphaera* sp. RUG761 [27] and *Mbb. boviskoreani* [38] both share the same ethanol-dependent methanogenesis capability.

## Conclusions

The work reported here has elucidated the main CH_3_-compound-forming pathways in the rumen and has identified the main bacteria that are involved. The ability to form methanol from methoxylated pectin via PME activity is widespread among rumen bacteria, but is most prevalent among members of the genus *Prevotella*. TMA release from plant-derived choline via TMA lyase activity is restricted to a much narrower spectrum of bacteria, principally *Olsenella* and *Caecibacter* in the ovine rumen and *Olsenella*, *Caecibacter*, and *Eubacterium* in the bovine rumen. The pectin enrichment experiment using sheep rumen contents has provided a unique insight into a specific example of a pectin-utilising and methane-forming consortium. As the techniques for assembling genomes from metagenomic sequencing data continue to improve, it is likely that more investigation of enrichment cultures and synthetic consortia will elucidate the complex relationships and inter-dependencies that occur in CH_3_-compound formation in the rumen. The screening work now allows the main CH_3_-compound-forming bacteria to be targeted specifically with the aim of finding ways to reduce their growth and/or enzymatic activities. By using such microbiological interventions we aim to reduce the supply of CH_3_-compound substrates to methanogens and thereby limit the amount of methane formed from by methylotrophic methanogens in the rumen.

## Methods

### Identification of TMA forming potential in rumen microbiome datasets

The Hidden Markov Model (HMM) profiles of CutC and CutD was kindly provided by Rath et al. [25]. The HMMER software package [46] using default cutoffs for CutD and a score cutoff of > 1500 for CutC was used to identify potential *cut* genes in the Hungate1000 Collection genomes [28], the rumen metagenome assembled genomes (MAGs) dataset [27] and the combined assembly of the High/Low dataset [26] and re-assembled (using SPADES) metagenome data of rumen microbial communities from low MY sheep (tags 1283, 1265, 1435, 1449 at 2 time points) used in the combined assembly of the High/Low dataset above. For phylogenetic alignment of the CutC genes, protein sequences were aligned using MUSCLE [47]. Maximum likelihood trees were constructed in MEGA7 [48] using the Le Gascuel 2008 method [49]. Statistical support for the tree was obtained by bootstrapping 100 iterations and the pyruvate-formate lyase gene from *Methanobrevibacter ruminantium* M1 (WP_012956318.1) [39] was used as the outgroup. A taxonomic classification of the CutC genes identified from the Rumen MAGs and the High/Low combined assembly datasets were assigned using the top blast hit result against the NCBI non-redundant (nr) protein database. An E-value cutoff of less than 1e-5 was used.

### Identification of potential pectinesterase (PME) activity in rumen microbiome datasets

The Hidden Markov Model (HMM) profile of PF01095 (PME domain) was downloaded from the Pfam database (http://pfam.sanger.ac.uk/), and HMMER software was used to detect the presence of PME genes using default cutoffs against the three datasets described above [26–28]. The taxonomy of the PME genes identified from the High/Low dataset were assigned using the top BLAST hit result against the NCBI non-redundant (nr) protein database, using an E-value cutoff of less than 1e-5.

### Read mapping to identified CutC and PME genes

Metagenomic and metatranscriptome reads of each of the high/low microbiome samples (see Additional file 5: Table S4A and as described previously [50]) were mapped to the identified rumen CutC (*n* = 18) and PME-containing genes (*n* = 2730) from the Hungate1000 and the high/low combined assembly using BBmap (https://sourceforge.net/projects/bbmap) with an ID cut-off of 98% sequence similarity. Results were summarised using Samtools version 1.9 [51], see Additional file 5: Table S4B. Read counts were normalised using reads per kilobase per million (RPKM).

### Pectin enrichment culture from sheep rumen contents

A pectin enrichment of the microbiota from sheep rumen contents was set up to assess the types of organisms capable of mediating pectin degradation coupled to methylotrophic methanogenesis. Rumen contents from sheep grazing a ryegrass-white clover pasture, were collected and filtered through 335 μm nylon mesh into Oakridge tubes which had been flushed with O_2_-free CO_2_. The tubes were centrifuged at low speed (200 x *g*) for 15 min at room temperature and the supernatant transferred to fresh tubes flushed with O_2_-free CO_2_. The tubes were centrifuged at 28,000 x *g* for 30 min at room temperature, the supernatant discarded, and the cell pellet was re-suspended in 5 mL of anaerobic RM02 base medium [52], then the volume taken up to 50 mL using the same medium. The tubes were centrifuged again at 28,000 x *g* for 30 min at room temperature, the supernatant discarded, and the cell pellet was re-suspended in 5 mL of anaerobic RM02 base medium under a stream of O_2_-free CO_2_. The re-suspended cells were 10-fold serially diluted into RM02 medium containing 1% pectin (Sigma apple pectin, poly-D-galacturonic acid methyl ester) and incubated at 39 °C. The gas composition of the headspace of each enrichment tube was monitored daily using gas chromatography [39] and when methane appeared, an aliquot of the culture was observed using phase contrast and fluorescence microscopy. The methane-producing enrichment tubes were dominated by fluorescent cocci, along with other non-fluorescent cells. Aliquots of methane-positive cultures were plated onto agar plates of RM02 medium containing 1% pectin inside an anaerobic chamber (Coy Laboratory Products, 96% CO_2_:4% H_2_ atmosphere) and incubated anaerobically in air-tight gas canisters at 39 °C until colonies formed. Single colonies were picked from plates inside the anaerobic chamber into fresh RM02-pectin broth medium and assessed for culture purity by PCR amplification using bacterial- and archaeal-specific 16S rRNA gene primers. One of the single-colony sub-cultures, designated MEC1, was found to contain a limited microbial diversity by phase contrast and fluorescent microscopy, and according to the 16S rRNA gene sequences retrieved from this culture, was dominated by two organisms; a methanogen associated with the genus *Methanosphaera* sp. and a bacterium affiliated with the family Sphaerochaetaceae.

### Metagenome sequencing and assembly of the pectin enrichment culture

Community genomic DNA was extracted from the limited diversity MEC1 metagenome and submitted for sequencing as part of the Hungate1000 project at the Joint Genome Institute [28]. Sequencing used Illumina HiSeq 2500-1 TB technology and the metagenome sequences were assembled into 107 contigs using SPAdes V 3.11.1 [53]. The 26 largest contigs, ranging in size from 1.49 kb to 796 Kb, were sorted into 3 bins using MetaBAT [54]. Each bin had a scaffold which contained an almost full length 16S rRNA gene sequence allowing their preliminary taxonomic identification (Additional file 4: Table S3). Genomes were annotated by the DOE–JGI genome annotation pipeline [55–58]. CheckM analysis [59] of the three assembled genomes was conducted to estimate their completeness and degree of contamination. The evolutionary relationship of the alcohol dehydrogenase and aldehyde dehydrogenase genes from Organism 3 (*Methanosphaera* sp.) MAG with similar genes from rumen methanogens were inferred using the Neighbor-Joining method [60]. The percentage of replicate trees in which the associated taxa clustered together in the bootstrap test (1000 replicates) are shown next to the branches [61]. The trees were drawn to scale, with branch lengths in the same units as those of the evolutionary distances used to infer the phylogenetic tree. The evolutionary distances were computed using the JTT matrix-based method [62] and the units are the number of amino acid substitutions per site. Evolutionary analyses were conducted in MEGA7 [48].

## Supplementary information

**Additional file 1: Table S1.** A. CutC genes in the combined metagenome/metatranscriptome and Hungate1000 datasets. B. Metagenome abundance of PME genes identified from the combined metagenome/metatranscriptome and Hungate1000 datasets. C. Metatranscriptome abundance of PME transcripts of genes identified from the combined metagenome/metatranscriptome and Hungate1000 datasets. D. Top Blast hit against the 505 Pfam01095 containing ORFs identified from the rumen MAG dataset (*n* = 913).

**Additional file 2: Figure S2.** Choline trimethylamine lyase and bacterial microcompartment gene synteny in SPADES re-assembled metagenomes of low MY sheep (A), and in bacterial genomes of rumen (B) or ruminant faecal origin (C) in the Hungate1000 Collection.

**Additional file 3: Table S2.** Pectin methyl esterase genes (Pfam01095) identified in the Hungate 1000 collection reference genome set

**Additional file 4: Table S3.** Contigs and genome sizes of the three organisms identified from the MEC1 limited diversity metagenome.

**Additional file 5: Table S4. **A Overview of samples analysed in this study. B Results of the mapping of the high/low sample reads to the identified rumen CutC and PME-containing genes from the Hungate1000 and the high/low combined assembly.

## Data Availability

The metagenome and metatranscriptome datasets used in this study are accessible at the National Centre for Biotechnology Information Sequence Read Archive (SRA; http://www.ncbi.nlm.nih.gov/sra) accession number SRA075938, BioProject number PRJNA202380, plus additional 16S rRNA gene amplicon sequence data under the SRA experiment accession numbers: SRX1079958 - SRX1079985. The Hungate1000 genomes are available from Joint Genome Institute’s Integrated Microbial Genomes and Microbiome Samples (IMG/M) which can be accessed at http://genome.jgi.doe.gov/. The raw sequence data and assembled genomes and proteomes from the 913 rumen uncultured genomes (RUG) and HiC rumen uncultured genomes (hRUG) are available the European Nucleotide Archive under project PRJEB21624. The SPADES assemblies of low methane yield sheep rumen microbial communities from New Zealand can be accessed via their IMG Database Project IDs: Sheep Tag 1265 (Gp0054682; Gp0053989), Sheep Tag 1283 (Gp0054684, Gp0054469); Sheep Tag 1435 (Gp0053990, Gp0054493), Sheep Tag 1494 (Gp0054822, Gp0054568).

## Abbreviations

CO_2_: Carbon dioxide
CTMAL: Choline trimethylamine lyase
CutC: Choline trimethylamine lyase
CutD: Choline trimethylamine lyase activator protein
GH: Glycosyl hydrolase family
H_2_: Hydrogen
HMM: Hidden Markov Model
MAG(s): Metagenome Assembled Genome(s)
PCW: Plant cell wall
PL: Pectin lyase
PMEs: Pectin methyl esterases
PUL: Polysaccharide utilization loci
PULDB: CAZy PUL database
TMA: Trimethylamine
TMAO: Trimethylamine-*N*-oxide
