# Supplementary material for: Occurrence and expression of genes encoding methyl-compound production in rumen bacteria
Source: Anim Microbiome. 2019 Nov 14;1:15. doi: 10.1186/s42523-019-0016-0 (PMC7807696; doi:10.1186/s42523-019-0016-0)
Supplement: Supplementary file 2 — Additional file 2: Figure S2. Choline trimethylamine lyase and bacterial microcompartment gene synteny in SPADES re-assembled metagenomes of low MY sheep (A), and in bacterial genomes of rumen (B) or ruminant faecal origin (C) in the Hungate1000 Collection. [file 42523_2019_16_MOESM2_ESM.pptx]

## Slide 1
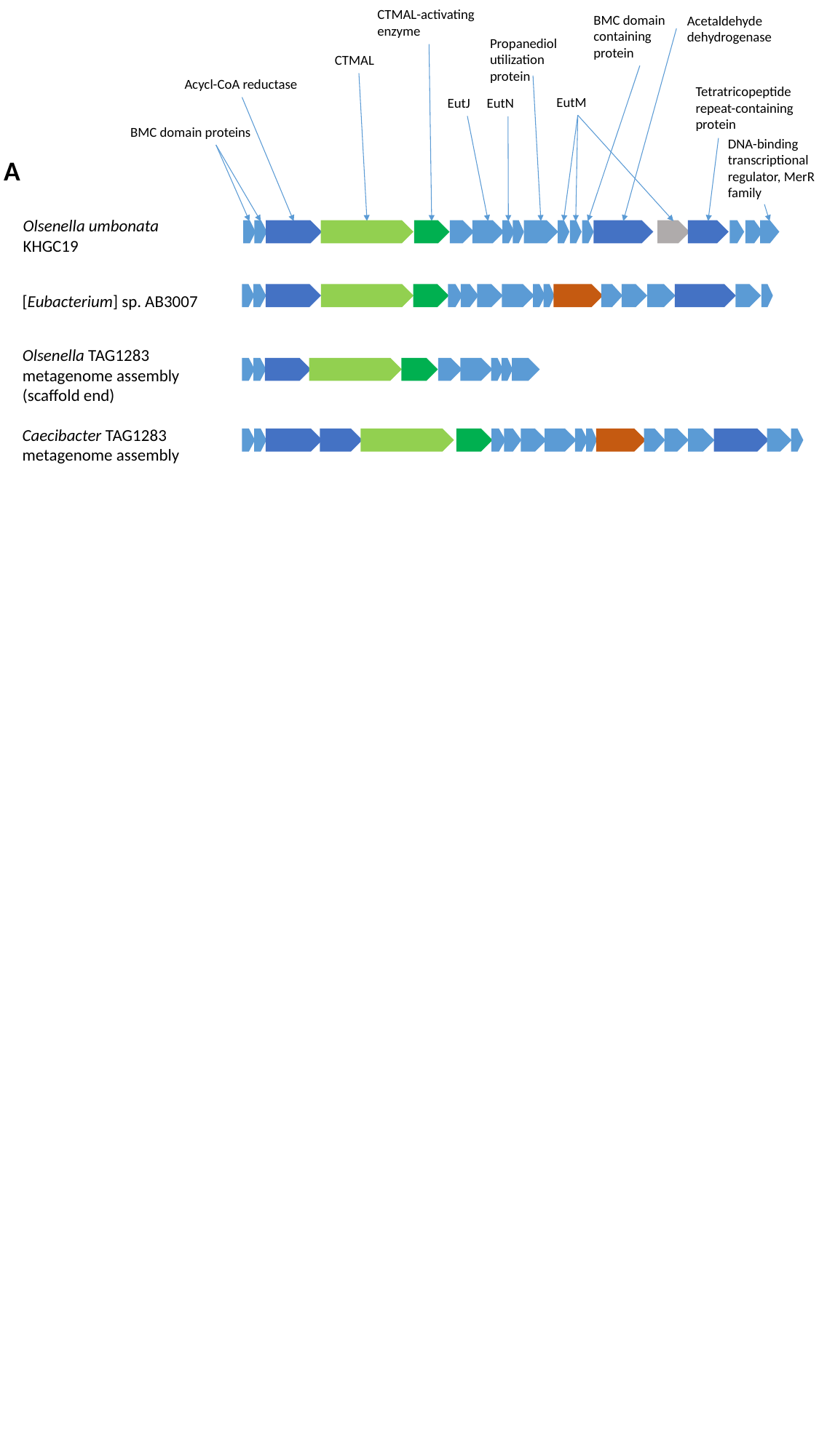

CTMAL-activating enzyme
BMC domain containing protein
Acetaldehyde dehydrogenase
Propanediol utilization protein
CTMAL
Acycl-CoA reductase
Tetratricopeptide repeat-containing protein
EutM
EutJ
EutN
BMC domain proteins
DNA-binding transcriptional regulator, MerR family
A
Olsenella umbonata KHGC19
[Eubacterium] sp. AB3007
Olsenella TAG1283 metagenome assembly (scaffold end)
Caecibacter TAG1283 metagenome assembly

## Slide 2
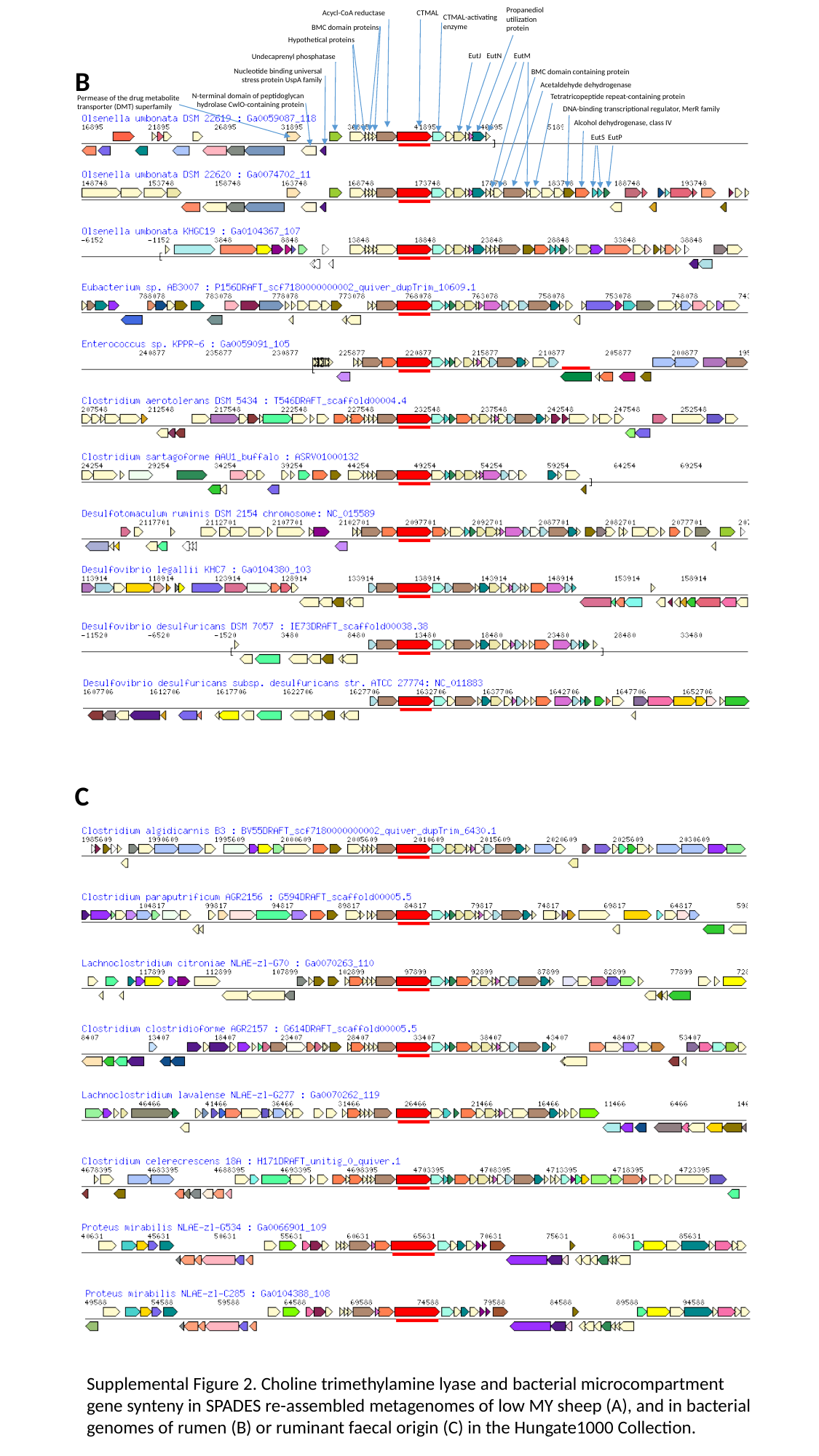

Propanediol utilization protein
CTMAL
Acycl-CoA reductase
CTMAL-activating enzyme
BMC domain proteins
Hypothetical proteins
EutM
EutJ
EutN
Undecaprenyl phosphatase
B
Nucleotide binding universal stress protein UspA family
BMC domain containing protein
Acetaldehyde dehydrogenase
N-terminal domain of peptidoglycan hydrolase CwlO-containing protein
Tetratricopeptide repeat-containing protein
Permease of the drug metabolite transporter (DMT) superfamily
DNA-binding transcriptional regulator, MerR family
Alcohol dehydrogenase, class IV
EutS
EutP
C
Supplemental Figure 2. Choline trimethylamine lyase and bacterial microcompartment gene synteny in SPADES re-assembled metagenomes of low MY sheep (A), and in bacterial genomes of rumen (B) or ruminant faecal origin (C) in the Hungate1000 Collection.
