# Supplementary material for: Occurrence and expression of genes encoding methyl-compound production in rumen bacteria
Source: Anim Microbiome. 2019 Nov 14;1:15. doi: 10.1186/s42523-019-0016-0 (PMC7807696; doi:10.1186/s42523-019-0016-0)
Supplement: Supplementary file 3 — Additional file 3: Table S2. Pectin methyl esterase genes (Pfam01095) identified in the Hungate 1000 collection reference genome set [file 42523_2019_16_MOESM3_ESM.docx]

| **Supplementary Table 2. Pectin methyl esterase genes (Pfam01095) identified in the Hungate 1000 collection reference genome set** | | | | | | | | | | |
| --- | --- | --- | --- | --- | --- | --- | --- | --- | --- | --- |
|  | |  |  |  | |  |  |  | | |
| **Strain** | **Bacterial species** | **ANI clique** | **IMG Gene ID** | **Locus tag** | | **aa** | **SP** | **Pfam** | | |
| **Bacteroidetes phylum** | |  |  |  | |  |  |  | | |
| KHT7 | *Bacteroidales* bacterium | 799 | 2657164777 | Ga0105823_1052 | | 306 |  |  | | |
|  |  |  | 2657164916 | Ga0105823_105141 | | 942 |  |  | | |
|  |  |  | 2657165859 | Ga0105823_11512 | | 1589 | SP | pfam13205 | | |
|  |  |  | 2657167031 | Ga0105823_13716 | | 345 | SP |  | | |
| Ga6A1 | *Bacteroidales* bacterium | 799 | 2562731972 | T544DRAFT_00234 | | 345 | SP |  | | |
|  |  |  | 2562732746 | T544DRAFT_01010 | | 939 |  |  | | |
|  |  |  | 2562732838 | T544DRAFT_01102 | | 1589 | SP | pfam13205 | | |
|  |  |  | 2562732982 | T544DRAFT_01247 | | 355 |  |  | | |
| Ga6A2 | *Bacteroidales* bacterium | 799 | 2562204427 | T538DRAFT_00474 | | 345 | SP |  | | |
|  |  |  | 2562205019 | T538DRAFT_01068 | | 939 |  |  | | |
|  |  |  | 2562205111 | T538DRAFT_01160 | | 1589 | SP | pfam13205 | | |
|  |  |  | 2562205256 | T538DRAFT_01306 | | 355 |  |  | | |
|  |  |  |  |  | |  |  |  | | |
| AR29 | *Bacteroides* sp*.* | 122 | 2595529655 | IE59DRAFT_00864 | | 322 | SP |  | | |
|  |  |  | 2595529656 | IE59DRAFT_00865 | | 577 |  | pfam13472 | | |
| KPPR-3 | *Bacteroides thetaiotaomicron* | 122 | 2595401683 | IE52DRAFT_01194 | | 322 | SP |  | | |
|  |  |  | 2595401684 | IE52DRAFT_01195 | | 577 |  | pfam13472 | | |
| NLAE-zl-C523 | *Bacteroides thetaiotaomicron* | 122 | 2513148748 | C523DRAFT_02466 | | 577 |  | pfam13472 | | |
|  |  |  | 2513148749 | C523DRAFT_02467 | | 322 | SP |  | | |
| NLAE-zl-G288 | *Bacteroides thetaiotaomicron* | 122 | 2657167519 | Ga0104385_101148 | | 322 | SP |  | | |
|  |  |  | 2657167520 | Ga0104385_101149 | | 577 |  | pfam13472 | | |
| NLAE-zl-C579 | *Bacteroides thetaiotaomicron* | 122 | 2514901975 | G288DRAFT_01438 | | 322 | SP |  | | |
|  |  |  | 2514901976 | G288DRAFT_01439 | | 577 |  | pfam13472 | | |
| KH3R12 | *Porphyromonadaceae* bacterium | 1672 | 2657045300 | Ga0105817_1235 | | 622 | SP | pfam07859 | | |
| NLAE-zl-C104 | *Porphyromonadaceae* bacterium | 1672 | 2657019456 | Ga0105827_10646 | | 622 | SP | pfam07859 | | |
| KHP3R9 | *Porphyromonadaceae* bacterium | 16789 | 2657057416 | Ga0105824_101442 | | 774 | SP |  | | |
|  |  |  | 2657057418 | Ga0105824_101444 | | 624 | SP | pfam07859 | | |
| B14 | *Prevotella bryantii* | 2204 | 648641568 | PBR_0733 | | 325 | SP |  | | |
|  |  |  | 648641800 | PBR_1508 | | 1462 | SP | pfam13205, 13472 | | |
|  |  |  | 648641885 | PBR_1568 | | 1064 |  | pfam00544 | | |
|  |  |  | 648642224 | PBR_1877 | | 1117 | SP | pfam13205 | | |
| C21a | *Prevotella bryantii* | 2204 | 2525491954 | G638DRAFT_00332 | | 1117 | SP | pfam13205 | | |
|  |  |  | 2525492103 | G638DRAFT_00481 | | 318 |  |  | | |
|  |  |  | 2525492292 | G638DRAFT_00670 | | 1116 | SP | pfam00544 | | |
|  |  |  | 2525494103 | G638DRAFT_02484 | | 1456 | SP | pfam13205, 13472 | | |
|  |  |  | 2525492482 ^1^ | G638DRAFT_00861 | | 1058 | SP |  | | |
| FB3001 | *Prevotella bryantii* | 2204 | 2624546741 | Ga0066874_105141 | | 1116 | SP | pfam00544 | | |
|  |  |  | 2624547233 | Ga0066874_10823 | | 1456 | SP | pfam13205, 13472 | | |
|  |  |  | 2624547416 | Ga0066874_10990 | | 318 |  |  | | |
|  |  |  | 2624547751 | Ga0066874_1131 | | 1117 | SP | pfam13205 | | |
| KHPX14 | *Prevotella bryantii* | 2204 | 2656975552 | Ga0104364_101458 | | 1117 | SP | pfam13205 | | |
|  |  |  | 2656975703 | Ga0104364_101609 | | 318 |  |  | | |
|  |  |  | 2656976187 | Ga0104364_10343 | | 1116 | SP | pfam00544 | | |
|  |  |  | 2656977696 | Ga0104364_11020 | | 1456 | SP | pfam13205, 13472 | | |
|  |  |  |  |  | |  |  |  | | |
| AR32 | *Prevotella ruminicola* | 1197 | 2657222365 | Ga0104419_0143 | | 324 | SP |  | | |
| KHP7 | *Prevotella* sp*.* | 1197 | 2608494206 | Ga0059113_1424 | | 324 | SP |  | | |
| P6B1 | *Prevotella* sp*.* | 767 | 2562537118 | T495DRAFT_0756 | | 326 | SP |  | | |
|  |  |  | 2562539216 | T495DRAFT_2860 | | 325 | SP |  | | |
| P6B4 | *Prevotella* sp*.* | 767 | 2558948617 | T491DRAFT_00491 | | 325 | SP |  | | |
|  |  |  | 2558949140 | T491DRAFT_01015 | | 326 | SP |  | | |
| 23 | *Prevotella ruminicola* | 2105 | 646735195 | PRU_0012 | | 324 | SP |  | | |
|  |  |  | 646736239 | PRU_1101 | | 517 | SP |  | | |
| BPI-34 | *Prevotella ruminicola* | 2105 | 2626518399 | Ga0070635_0741 | | 501 | SP |  | | |
|  |  |  | 2626520343 | Ga0070635_2691 | | 324 | SP |  | | |
| BPI-162 | *Prevotella ruminicola* | 2105 | 2624628911 | Ga0070636_102255 | | 501 | SP |  | | |
|  |  |  | 2624629858 | Ga0070636_105151 | | 324 | SP |  | | |
| RM4 | *Prevotella ruminicola* | 2105 | 2562750886 | T499DRAFT_1422 | | 432 |  |  | | |
|  |  |  | 2562751765 | T499DRAFT_2304 | | 324 | SP |  | | |
|  |  |  | 2562751842 | T499DRAFT_2382 | | 501 | SP |  | | |
| KHP1 | *Prevotella ruminicola* | 2105 | 2608636975 | Ga0059112_0428 | | 501 | SP |  | | |
|  |  |  | 2608639265 | Ga0059112_2718 | | 324 | SP |  | | |
| BPI-145 | *Prevotella* sp*.* | 1318 | 2624732858 | Ga0066893_101166 | | 354 | SP |  | | |
|  |  |  | 2624733151 | Ga0066893_102160 | | 323 | SP |  | | |
|  |  |  | 2624733566 | Ga0066893_104122 | | 1674 |  | pfam13205 | | |
|  |  |  | 2624733943 | Ga0066893_10751 | | 1052 | SP |  | | |
| BPI-148 | *Prevotella* sp*.* | 1318 | 2624720943 | Ga0066894_10376 | | 1674 |  | pfam13205 | | |
|  |  |  | 2624721118 | Ga0066894_10452 | | 1052 | SP |  | | |
|  |  |  | 2624721338 | Ga0066894_10571 | | 323 | SP |  | | |
|  |  |  | 2624722647 | Ga0066894_11745 | | 354 | SP |  | | |
| ATCC 19189 | *Prevotella ruminicola* | 10078 | 2597417205 | EI35DRAFT_1097 | | 501 | SP |  | | |
|  |  |  | 2597418365 | EI35DRAFT_2259 | | 324 | SP |  | | |
| D31d | *Prevotella ruminicola* | 16185 | 2657194672 | Ga0104403_0255 | | 432 |  |  | | |
|  |  |  | 2657195439 | Ga0104403_1023 | | 501 | SP |  | | |
|  |  |  | 2657196402 | Ga0104403_1987 | | 374 | SP |  | | |
|  |  |  | 2657197425 | Ga0104403_3013 | | 324 | SP |  | | |
| Ga6B6 | *Prevotella ruminicola* | 7984 | 2558938062 | T500DRAFT_00470 | | 432 |  |  | | |
|  |  |  | 2558938675 | T500DRAFT_01084 | | 329 | SP |  | | |
| KHT3 | *Prevotella ruminicola* | 16812 | 2657154961 | Ga0104360_10775 | | 374 | SP |  | | |
|  |  |  | 2657156886 | Ga0104360_12812 | | 324 | SP |  | | |
| FD3004 | *Prevotella* sp*.* | 8630 | 2574376211 | T325DRAFT_2485 | | 324 | SP |  | | |
|  |  |  |  |  | |  |  |  | | |
| MA2016 | *Prevotella* sp*.* | 8090 | 2561495551 | T360DRAFT_1900 | | 330 | SP |  | | |
|  |  |  |  |  | |  |  |  | | |
| NE3005 | *Prevotella* sp*.* | 13007 | 2624539847 | Ga0066875_101561 | | 322 | SP |  | | |
| TC2-28 | *Prevotella* sp*.* | 13049 | 2624894211 | Ga0066886_101173 | | 886 |  |  | | |
|  |  |  | 2624895418 | Ga0066886_10615 | | 362 | SP |  | | |
|  |  |  | 2624895419 | Ga0066886_10616 | | 354 |  |  | | |
|  |  |  | 2624895675 | Ga0066886_107111 | | 700 | SP | pfam09492 | | |
|  |  |  | 2624896165 | Ga0066886_11126 | | 328 | SP |  | | |
| AGR2160 | *Prevotella* sp*.* | 5559 | 2525495296 | G604DRAFT_00997 | | 1463 | SP | pfam13205, 13472 | | |
|  |  |  | 2525495316 | G604DRAFT_01017 | | 305 | SP |  | | |
|  |  |  | 2525495608 | G604DRAFT_01310 | | 1064 | SP | pfam09479 | | |
| TF2-5 | *Prevotella* sp*.* | 13008 | 2624542843 | Ga0066888_10237 | | 1846 | SP | pfam09479 | | |
|  |  |  | 2624543961 | Ga0066888_10612 | | 823 | SP |  | | |
|  |  |  | 2624544268 | Ga0066888_107122 | | 325 | SP |  | | |
|  |  |  | 2624544341 | Ga0066888_10842 | | 1688 | SP | pfam13205, 13472 | | |
| NLAE-zl-C11 | *Bacteroides ovatus* | 2581 | 2513122929 | C11DRAFT_00307 | | 451 | SP |  | | |
|  |  |  | 2513122945 | C11DRAFT_00323 | | 579 |  | pfam13472 | | |
|  |  |  | 2513122946 | C11DRAFT_00324 | | 323 |  |  | | |
|  |  |  | 2513123222 | C11DRAFT_00600 | | 434 | SP |  | | |
|  |  |  | 2513123227 | C11DRAFT_00605 | | 539 |  | pfam13205 | | |
| NLAE-zl-C34 | *Bacteroides ovatus* | 2581 | 2513142110 | C34DRAFT_00533 | | 451 | SP |  | | |
|  |  |  | 2513142126 | C34DRAFT_00549 | | 579 |  | pfam13472 | | |
|  |  |  | 2513142127 | C34DRAFT_00550 | | 323 |  |  | | |
|  |  |  | 2513143373 | C34DRAFT_01796 | | 434 | SP |  | | |
|  |  |  | 2513143378 | C34DRAFT_01801 | | 539 |  | pfam13205 | | |
| NLAE-zl-C57 | *Bacteroides ovatus* | 2581 | 2657075512 | Ga0104390_101241 | | 323 |  |  | | |
|  |  |  | 2657075513 | Ga0104390_101242 | | 579 |  | pfam13472 | | |
|  |  |  | 2657075529 | Ga0104390_101258 | | 451 | SP |  | | |
|  |  |  | 2657075827 | Ga0104390_101660 | | 539 |  | pfam13205 | | |
|  |  |  | 2657075832 | Ga0104390_101665 | | 434 | SP |  | | |
| NLAE-zl-C500 | *Bacteroides ovatus* | 2581 | 2657059513 | Ga0104402_1003144 | | 539 |  | pfam13205 | | |
|  |  |  | 2657059518 | Ga0104402_1003149 | | 434 | SP |  | | |
|  |  |  | 2657059593 | Ga0104402_100457 | | 804 |  | pfam13149 | | |
|  |  |  | 2657059607 | Ga0104402_100471 | | 542 | SP |  | | |
|  |  |  | 2657059613 | Ga0104402_100477 | | 579 |  | pfam13472 | | |
|  |  |  | 2657059614 | Ga0104402_100478 | | 323 | SP |  | | |
| NLAE-zl-G202 | *Bacteroides vulgatus* | 1800 | 2624770164 | Ga0066897_101113 | | 316 | SP |  | | |
|  |  |  | 2624770169 | Ga0066897_101118 | | 506 |  |  | | |
|  |  |  | 2624770181 | Ga0066897_101130 | | 575 | SP | pfam13472 | | |
|  |  |  | 2624770773 | Ga0066897_10184 | | 360 | SP | pfam13472 | | |
| NLAE-zl-G339 | *Bacteroides xylanisolvens* | 2064 | 2624747705 | Ga0066898_12530 | | 323 | SP |  | | |
|  |  |  | 2624747706 | Ga0066898_12531 | | 582 |  | pfam13472 | | |
|  |  |  | 2624747724 | Ga0066898_12549 | | 803 |  | pfam13149 | | |
| NLAE-zl-C202 | *Bacteroides xylanisolvens* | 2064 | 2657172919 | Ga0104384_10330 | | 323 | SP |  | | |
|  |  |  | 2657172920 | Ga0104384_10331 | | 582 |  | pfam13472 | | |
|  |  |  | 2657172938 | Ga0104384_10349 | | 803 |  | pfam13149 | | |
| NLAE-zl-C29 | *Bacteroides xylanisolvens* | 2064 | 2513133462 | C29DRAFT_01456 | | 323 | SP |  | | |
|  |  |  | 2513133463 | C29DRAFT_01457 | | 582 |  | pfam13472 | | |
|  |  |  | 2513133469 | C29DRAFT_01463 | | 547 | SP |  | | |
| NLAE-zl-C182 | *Bacteroides xylanisolvens* | 2064 | 2513128130 | C182DRAFT_00875 | | 803 |  | pfam13149 | | |
|  |  |  | 2513128148 | C182DRAFT_00893 | | 582 |  | pfam13472 | | |
|  |  |  | 2513128149 | C182DRAFT_00894 | | 323 | SP |  | | |
| NLAE-zl-C339 | *Bacteroides xylanisolvens* | 2064 | 2513137748 | C339DRAFT_00910 | | 323 | SP |  | | |
|  |  |  | 2513137748 | C339DRAFT_00911 | | 582 |  | pfam13472 | | |
|  |  |  | 2513137767 | C339DRAFT_00929 | | 803 |  | pfam13149 | | |
| NLAE-zl-G310 | *Bacteroides xylanisolvens* | 2064 | 2514906187 | G310DRAFT_00523 | | 323 | SP |  | | |
|  |  |  | 2514906188 | G310DRAFT_00524 | | 582 |  | pfam13472 | | |
|  |  |  | 2514906206 | G310DRAFT_00542 | | 803 |  | pfam13149 | | |
| NLAE-zl-G421 | *Bacteroides xylanisolvens* | 2064 | 2514910815 | G421DRAFT_00218 | | 803 |  | pfam13149 | | |
|  |  |  | 2514910833 | G421DRAFT_00236 | | 582 |  | pfam13472 | | |
|  |  |  | 2514910834 | G421DRAFT_00237 | | 323 | SP |  | | |
| **Fimicutes phylum** | |  |  |  | |  |  |  | | |
| VTM3R78 | *Bacillus licheniformis* | 1162 | 2624512341 | Ga0070258_102235 | | 317 |  |  | | |
| AAU1 | *Bacillus nealsonii* | 6654 | 2543438630 | A499_23662 | | 317 |  |  | | |
| AGR2140 | *Clostridium butyricum* | 1870 | 2525473467 | G607DRAFT_00324 | | 1369 | SP | pfam01473 | | |
| B6405 | *Clostridium* sp*.* | 13477 | 2626506351 | Ga0070257_103013 | | 1195 | SP |  | | |
|  |  |  | 2626506352 | Ga0070257_103014 | | 1203 | SP |  | | |
| C2 | *Enterococcus mundtii* | 546 | 2608584062 | Ga0007610_1183 | | 301 |  |  | | |
| NLAE-zl-G268 | *Enterococcus casseliflavus* | 2155 | 2624610360 | Ga0070259_102134 | | 305 |  |  | | |
| NLAE-zl-C414 | *Enterococcus casseliflavus* | 2155 | 2654389421 | Ga0104391_102198 | | 305 |  |  | | |
| NK3D112 | *Erysipelotrichaceae* bacterium | 8275 | 2563355317 | T373DRAFT_01338 | | 283 |  |  | | |
| MD2001 | *Butyrivibrio fibrisolvens* | 317 | 2526132240 | G635DRAFT_00036 | | 285 |  |  | | |
|  |  |  | 2526132636 | G635DRAFT_00435 | | 1556 | SP |  | | |
|  |  |  | 2526134809 | G635DRAFT_02617 | | 2153 | SP | pfam01473, 02368 | | |
| AB2020 | *Butyrivibrio fibrisolvens* | 317 | 2526107090 | G616DRAFT_00249 | | 285 |  |  | | |
|  |  |  | 2526108800 | G616DRAFT_01969 | | 1557 | SP |  | | |
|  |  |  | 2526109246 | G616DRAFT_02417 | | 2153 | SP | pfam01473, 02368 | | |
| FE2007 | *Butyrivibrio fibrisolvens* | 317 | 2525481101 | G624DRAFT_01214 | | 285 |  |  | | |
|  |  |  | 2525481251 | G624DRAFT_01364 | | 2153 | SP | pfam01473, 02368 | | |
|  |  |  | 2525483354 | G624DRAFT_03475 | | 1552 | SP |  | | |
| ND3005 | *Butyrivibrio fibrisolvens* | 317 | 2526123315 | G615DRAFT_01262 | | 285 |  |  | | |
|  |  |  | 2526124448 | G615DRAFT_02403 | | 2153 | SP | pfam01473, 02368 | | |
|  |  |  | 2526124899 | G615DRAFT_02856 | | 1534 | SP |  | | |
| YRB2005 | *Butyrivibrio fibrisolvens* | 317 | 2525484169 | G591DRAFT_00545 | | 1595 | SP |  | | |
|  |  |  | 2525485390 | G591DRAFT_01769 | | 2153 | SP | pfam01473, 02368 | | |
|  |  |  | 2525486821 | G591DRAFT_03206 | | 285 |  |  | | |
| WTE3004 | *Butyrivibrio fibrisolvens* | 317 | 2525488613 | G623DRAFT_00994 | | 2153 | SP | pfam01473, 02368 | | |
|  |  |  | 2525489885 | G623DRAFT_02268 | | 1563 | SP |  | | |
|  |  |  | 2525491147 | G623DRAFT_03539 | | 285 |  |  | | |
| AR40 | *Butyrivibrio fibrisolvens* | 317 | 2624760626 | Ga0070254_11337 | | 285 |  |  | | |
|  |  |  | 2624761235 | Ga0070254_11921 | | 2153 | SP | pfam01473, 02368 | | |
|  |  |  | 2624761774 | Ga0070254_12536 | | 1532 | SP |  | | |
| TB | *Butyrivibrio fibrisolvens* | 317 | 2595459547 | IE63DRAFT_00394 | | 285 |  |  | | |
|  |  |  | 2595460950 | IE63DRAFT_01802 | | 1551 | SP |  | | |
|  |  |  | 2595461596 | IE63DRAFT_02452 | | 2148 | SP | pfam01473, 02368 | | |
| D1 | *Butyrivibrio fibrisolvens* | 9437 | 2587776792 | EJ22DRAFT_01989 | | 283 |  |  | | |
|  |  |  | 2587778818 | EJ22DRAFT_04032 | | 2089 | SP | pfam01473 | | |
|  |  |  | 2587778865 | EJ22DRAFT_04079 | | 1505 | SP |  | | |
| B316 | *Butyrivibrio proteoclasticus* | 2176 | 648131804 | bpr_I1084 | | 341 |  |  | | |
|  |  |  | 648133203 | bpr_I2473 | | 2732 | SP | pfam01473 | | |
| FD2007 | *Butyrivibrio proteoclasticus* | 2176 | 2558986242 | T326DRAFT_00433 | | 2732 | SP | pfam01473 | | |
|  |  |  | 2558988463 | T326DRAFT_02661 | | 336 |  |  | | |
| P6B7 | *Butyrivibrio proteoclasticus* | 7987 | 2558951463 | T541DRAFT_00300 | | 2472 | SP |  | | |
|  |  |  | 2558953307 | T541DRAFT_02149 | | 311 |  |  | | |
| P18 | *Butyrivibrio proteoclasticus* | 13032 | 2624756275 | Ga0066879_11030 | | 334 |  |  | | |
| AC2005 | *Butyrivibrio* sp*.* | 5552 | 2525435289 | G602DRAFT_03732 | | 327 |  |  | | |
| AE2015 | *Butyrivibrio* sp*.* | 5657 | 2526103693 | G585DRAFT_00127 | | 334 |  |  | | |
| AE2032 | *Butyrivibrio* sp*.* | 8805 | 2579734343 | BO18DRAFT_0652 | | 2755 | SP |  | | |
|  |  |  | 2579735309 | BO18DRAFT_1618 | | 379 |  |  | | |
| AE3004 | *Butyrivibrio* sp*.* | 8227 | 2562668890 | BV60DRAFT_3765 | | 327 |  |  | | |
| AE3006 | *Butyrivibrio* sp*.* | 309 | 2525412063 ^2^ | G603DRAFT_03520 | | 327 |  |  | | |
| AE3009 | *Butyrivibrio* sp*.* | 5675 | 2526207827 | G588DRAFT_00971 | | 3005 | SP |  | | |
|  |  |  | 2526209047 | G588DRAFT_02196 | | 1059 | SP | pfam00553, 09492, 14200 | | |
|  |  |  | 2526210322 | G588DRAFT_03477 | | 325 |  |  | | |
| FC2001 | *Butyrivibrio* sp*.* | 313 | 2526149928 | G601DRAFT_03414 | | 329 |  |  | | |
| XPD2002 | *Butyrivibrio* sp*.* | 313 | 2525439134 | G587DRAFT_02812 | | 329 |  |  | | |
| FCS014 | *Butyrivibrio* sp*.* | 6690 | 2545782737 | T456DRAFT_02153 | | 2415 |  |  | | |
|  |  |  | 2545782768 | T456DRAFT_02184 | | 353 |  |  | | |
|  |  |  | 2545784286 | T456DRAFT_03707 | | 321 |  |  | | |
|  |  |  | 2545784200 | T456DRAFT_03621 | |  |  |  | | |
| M55 | *Butyrivibrio* sp*.* |  | 2656998728 | Ga0104398_11115 | |  |  |  | | |
| INlla14 | *Butyrivibrio* sp*.* | 9791 | 2595394791 | IE62DRAFT_00691 | | 323 |  |  | | |
|  |  |  | 2595394961 | IE62DRAFT_00863 | | 2640 | SP | pfam01473 | | |
| MC2021 | *Butyrivibrio* sp*.* | 7910 | 2558261963 | T359DRAFT_01678 | | 324 |  |  | | |
|  |  |  | 2558262500 | T359DRAFT_02216 | | 3452 | SP | pfam13290 | | |
|  |  |  | 2558262833 | T359DRAFT_02552 | | 2485 | SP | pfam01473 | | |
|  |  |  | 2558263764 | T359DRAFT_03488 | | 1048 | SP | pfam00553, 09492, 14200 | | |
| NC2007 | *Butyrivibrio* sp*.* | 314 | 2525441767 | G632DRAFT_01393 | | 393 |  |  | | |
|  |  |  | 2525442883 | G632DRAFT_02513 | | 2810 | SP |  | | |
| FCS006 | *Butyrivibrio* sp*.* | 314 | 2526245483 | G633DRAFT_0518 | | 2816 | SP |  | | |
|  |  |  | 2526246119 | G633DRAFT_1155 | | 388 |  |  | | |
| NC3005 | *Butyrivibrio* sp*.* | 5561 | 2525504367 | G634DRAFT_00859 | | 1753 | SP |  | | |
| OB235 | *Butyrivibrio* sp*.* | 311 | 2608486034 ^3^ | Ga0059103_13419 | | 327 |  |  | | |
| Su6 | *Butyrivibrio* sp*.* | 9882 | 2596380070 | IE64DRAFT_02500 | | 2616 | SP |  | | |
|  |  |  | 2596380151 | IE64DRAFT_02582 | | 334 |  |  | | |
| VCB2001 | *Butyrivibrio* sp*.* | 5563 | 2525512657 | G589DRAFT_02215 | | 381 |  |  | | |
|  |  |  | 2525513456 | G589DRAFT_03016 | | 2813 | SP |  | | |
| VCB2006 | *Butyrivibrio* sp*.* | 5564 | 2525516893 | G613DRAFT_00449 | | 356 |  |  | | |
|  |  |  | 2525517818 | G613DRAFT_01380 | | 2730 | SP | pfam01473 | | |
| VCD2006 | *Butyrivibrio* sp*.* | 5550 | 2525422885 | G586DRAFT_03300 | | 324 |  |  | | |
| XBB1001 | *Butyrivibrio* sp*.* | 5549 | 2525413905 | G631DRAFT_01704 | | 2640 | SP | pfam01473 | | |
|  |  |  | 2525414787 | G631DRAFT_02589 | | 323 |  |  | | |
| XPD2006 | *Butyrivibrio* sp*.* | 5562 | 2525508411 | G590DRAFT_01489 | | 2913 | SP |  | | |
|  |  |  | 2525510102 | G590DRAFT_03186 | | 358 |  |  | | |
| YAB3001 | *Butyrivibrio* sp*.* | 9886 | 2596390955 | IE68DRAFT_00976 | | 349 |  |  | | |
| H1 | *Cellulosilyticum ruminicola* | 17245 | 2667997859 | Ga0118697_100217 | | 327 |  |  | | |
|  |  |  | 2667999177 | Ga0118697_101440 | | 1673 | SP | pfam00395, 00544, 09479 | | |
| FB3002 | *Eubacterium ruminantium* | 13023 | 2624633296 | Ga0066876_11148 | | 267 |  |  | | |
| NK4B19 | *Lachnobacterium bovis* | 319 | 2525515362 | G620DRAFT_01290 | | 1335 | SP |  | | |
| AE2004 | *Lachnobacterium bovis* | 319 | 2561418896 | CC91DRAFT_00876 | | 1309 | SP |  | | |
| C6A12 | *Lachnobacterium bovis* | 319 | 2558942320 | T535DRAFT_01836 | | 1311 | SP |  | | |
| S1b | *Lachnobacterium bovis* | 319 | 2595182312 | IE07DRAFT_00255 | | 1289 | SP |  | | |
| YZ 87 | *Lachnobacterium bovis* | 9742 | 2595181112 | IE72DRAFT_01552 | | 1309 | SP |  | | |
| X8A62 | *Lachnoclostridium aerotolerans* | 7989 | 2558961965 | T546DRAFT_00996 | | 321 |  |  | | |
|  |  |  | 2558963694 | T546DRAFT_02733 | | 1993 | SP | pfam00544, 01473 | | |
| 18A | *Lachnoclostridium celerecrescens* | 9874 | 2596301580 | H171DRAFT_1425 | | 321 |  |  | | |
| T90 | *Lachnoclostridium clostridioforme* | 31 | 2595146311 | IE41DRAFT_00168 | | 352 |  |  | | |
| NLAE-zl-G208 | *Lachnoclostridium clostridioforme* | 31 | 2657071157 | Ga0104393_102414 | | 352 |  |  | | |
| NLAE-zl-C196 | *Lachnoclostridium clostridioforme* | 31 | 2657063926 | Ga0104372_100237 | | 352 |  |  | | |
| D32 | *Lachnospira multipara* | 325 | 2525871284 | G600DRAFT_00601 | | 317 |  |  | | |
|  |  |  | 2525871674 | G600DRAFT_00991 | | 1534 |  |  | | |
|  |  |  | 2525872235 | G600DRAFT_01556 | | 2036 | SP | pfam13472 | | |
|  |  |  | 2525872342 | G600DRAFT_01664 | | 1961 |  |  | | |
| D15d | *Lachnospira multipara* | 325 | 2654381295 | Ga0104352_101281 | | 317 |  |  | | |
|  |  |  | 2654381601 | Ga0104352_10351 | | 1521 |  |  | | |
|  |  |  | 2654382663 | Ga0104352_11018 | | 1936 |  |  | | |
|  |  |  | 2654383252 | Ga0104352_11651 | | 2035 | SP | pfam13472 | | |
| LB2003 | *Lachnospira multipara* | 768 | 2562628954 | T537DRAFT_00178 | | 317 |  |  | | |
|  |  |  | 2562629952 | T537DRAFT_01178 | | 1512 |  |  | | |
|  |  |  | 2562630921 | T537DRAFT_02153 | | 2027 | SP | pfam13472 | | |
|  |  |  | 2562631079 | T537DRAFT_02314 | | 1881 |  |  | | |
| MC2003 | *Lachnospira multipara* | 768 | 2558973201 | T520DRAFT_00325 | | 1495 |  |  | | |
|  |  |  | 2558973576 | T520DRAFT_00700 | | 2029 | SP | pfam13472 | | |
|  |  |  | 2558974232 | T520DRAFT_01361 | | 1921 |  |  | | |
|  |  |  | 2558974592 | T520DRAFT_01724 | | 317 |  |  | | |
| M83 | *Lachnospira pectinoschiza* | 16876 | 2658526265 | Ga0104397_0016 | | 2045 | SP | pfam13472 | | |
|  |  |  | 2658527360 | Ga0104397_1111 | | 317 |  |  | | |
|  |  |  | 2658527874 | Ga0104397_1625 | | 1501 |  |  | | |
|  |  |  | 2658528578 | Ga0104397_2329 | | 1939 |  |  | | |
| NK3A20 | *Lachnospiraceae* bacterium | 9749 | 2595200957 | G595DRAFT_00081 | | 253 |  |  | | |
|  |  |  |  |  | |  |  |  | | |
| NK4A144 | *Lachnospiraceae* bacterium | 5556 | 2525467579 | G619DRAFT_01212 | | 1524 | SP |  | | |
| AB2028 | *Lachnospiraceae* bacterium | 7995 | 2558989928 | T322DRAFT_00776 | | 251 |  |  | | |
| MA2020 | *Lachnospiraceae* bacterium | 8530 | 2571113263 | T348DRAFT_00242 | | 1635 | SP |  | | |
| MC2017 | *Lachnospiraceae* bacterium | 8196 | 2562249042 | T350DRAFT_03730 | | 1637 | SP |  | | |
| ND2006 | *Lachnospiraceae* bacterium | 806 | 2562569970 | T521DRAFT_02724 | | 1705 | SP |  | | |
| KH1T2 | *Lachnospiraceae* bacterium | 806 | 2657159650 | Ga0105812_11339 | | 1701 | SP |  | | |
| C6A11 | *Lachnospiraceae* bacterium | 8166 | 2562058976 | T542DRAFT_0039 | | 269 |  |  | | |
| JC7 | *Lachnospiraceae* bacterium | 4078 | 2510833732 | LacJC7draft1_02036 | | 347 |  |  | | |
| C7 | *Lachnospiraceae* bacterium | 11347 | 2608668027 | Ga0007612_1462 | | 1300 | SP |  | | |
| KH1P17 | *Lachnospiraceae* bacterium | 11302 | 2608394690 | Ga0059119_104219 | | 1547 | SP |  | | |
| NK2B42 | *Oribacterium* sp*.* | 5546 | 2525393480 | G625DRAFT_01583 | | 388 |  |  | | |
| FC2011 | *Oribacterium* sp*.* | 801 | 2562242803 | T357DRAFT_01095 | | 386 |  |  | | |
| KHPX15 | *Oribacterium* sp*.* | 801 | 2657372516 | Ga0105813_11622 | | 399 |  |  | | |
| P6A1 | *Oribacterium* sp*.* | 8188 | 2562200884 | T515DRAFT_00210 | | 361 |  |  | | |
| WCC10 | *Oribacterium* sp*.* | 16655 | 2654566211 | Ga0105810_11244 | | 406 |  |  | | |
| AGR63 | *Lactobacillus mucosae* | 24 | 2585053808 ^4^ | BV56DRAFT_0334 | | 1309 |  | pfam01473 | | |
| AD2013 | *Ruminococcus albus* | 493 | 2540923606 | N773DRAFT_0571 | | 1124 | SP | pfam09492 | | |
| SY3 | *Ruminococcus albus* | 493 | 2579386752 | RASY3_17675 | | 1124 | SP | pfam09492 | | |
| AR67 | *Ruminococcus albus* | 9738 | 2595164932 | IE33DRAFT_01278 | | 1124 | SP | pfam09492 | | |
| KH2T6 | *Ruminococcus albus* | 16654 | 2654562794 | Ga0104370_10874 | | 1126 | SP | pfam09492 | | |
| 7 | *Ruminococcus albus* | 2245 | 649835138 | Rumal_1079 | | 1121 | SP | pfam09492 | | |
|  |  |  | 649836236 | Rumal_2200 | | 304 |  |  | | |
| 8 | *Ruminococcus albus* | 8255 | 2563187287 | CUS_7181 | | 304 |  |  | | |
|  |  |  | 2563187402 | CUS_5047 | | 596 | SP |  | | |
|  |  |  | 2563187778 | CUS_5668 | | 1095 | SP | pfam09492 | | |
| AE3010 | *Ruminococcus flavefaciens* | 6588 | 2541039114 | N774DRAFT_2680 | | 1145 | SP | pfam00544, DOC | | |
| MA2007 | *Ruminococcus flavefaciens* | 7926 | 2558353091 | T497DRAFT_00860 | | 1152 | SP | pfam00544, DOC | | |
| MC2020 | *Ruminococcus flavefaciens* | 8243 | 2562813674 | T490DRAFT_0797 | | 1153 | SP | pfam00544, DOC | | |
| ND2009 | *Ruminococcus flavefaciens* | 7919 | 2558327575 | T488DRAFT_00824 | | 1131 |  | pfam00544, DOC | | |
| YAD2003 | *Ruminococcus flavefaciens* | 9736 | 2595140747 | IE35DRAFT_00920 | | 1136 |  | pfam00544, DOC | | |
| YRD2003 | *Ruminococcus flavefaciens* | 9737 | 2595144116 | IE38DRAFT_01089 | | 1152 | SP | pfam00544, DOC | | |
| YL228 | *Ruminococcus flavefaciens* | 9802 | 2595443770 | IE39DRAFT_0994 | | 1144 | SP | pfam00544, DOC | | |
| SAb67 | *Ruminococcus flavefaciens* | 9732 | 2595117494 | IE37DRAFT_02530 | | 1040 |  | pfam00544, DOC | | |
| XPD3002 | *Ruminococcus flavefaciens* | 11315 | 2608470546 | Ga0059109_106149 | | 1136 | SP | pfam00544, DOC | | |
| Y1 | *Ruminococcus flavefaciens* | 13019 | 2624589893 | Ga0066885_104202 | | 1140 |  | pfam00544, DOC | | |
| C94 | *Ruminococcus flavefaciens* | 6819 | 2546898203 | L870DRAFT_02387 | | 1137 | SP | pfam00544, DOC | | |
| FD-1 | *Ruminococcus flavefaciens* | 3054 | 646115658 | RflaF_010100011634 | | 1154 | SP | pfam00544, DOC | | |
| 17 | *Ruminococcus flavefaciens* | 616 | 2548637603 | RUMFLA17DRAFT_03309 | | 587 | SP |  | | |
| 007c | *Ruminococcus flavefaciens* | 616 | 2573552439 | RF007C_08120 | | 1135 |  | pfam00544, DOC | | |
| HUN007 | *Ruminococcus* sp*.* | 8393 | 2566069261 | CC97DRAFT_1256 | | 945 | SP | pfam00544, DOC | | |
| YE71 | *Ruminococcus* sp*.* | 1078 | 2595157060 | IE40DRAFT_02657 | | 1120 | SP | pfam09492 | | |
| YE78 | *Ruminococcus* sp*.* | 1078 | 2595162547 | IE43DRAFT_02643 | | 1120 | SP | pfam09492 | | |
|  |  |  |  |  | |  |  |  | | |
| **Fibrobacteres phylum** | |  |  |  | |  |  |  | | |
| HM2 | *Fibrobacter succinogenes* subsp*. elongatus* | 10004 | 2597063017 | IE02DRAFT_2515 | | 687 |  |  | | |
| S85 | *Fibrobacter succinogenes* | 2078 | 646368992 | Fisuc_0679 | | 699 | SP |  | | |
|  |  |  |  |  | |  |  |  | | |
| **Spirochaetes phylum** | |  |  |  | |  |  |  | | |
| PB | *Treponema saccharophilum* | 3992 | 2509550514 | TresaDRAFT_1051 | | 1296 | SP | pfam13472 | | |
|  |  |  | 2509551562 | TresaDRAFT_2099 | | 683 | SP |  | | |
|  |  |  | 2509551592 | TresaDRAFT_2129 | | 328 |  |  | | |
|  |  |  |  |  | |  |  |  | | |
| **Proteobacteria phylum** | |  |  |  | |  |  |  | | |
| BR | *Cellvibrio* sp. | 6495 | 2539678523 | O59_000294 | | 371 |  |  | | |
|  |  |  | 2539678770 | O59_000541 | | 1117 | SP |  | | |
|  |  |  | 2539681087 | O59_000541 | | 1462 | SP | pfam13205 | | |
| KPR-6 | *Enterobacter* sp. | 11398 | 2609053252 | Ga0059124_1264 | | 427 | SP |  | | |
|  |  |  | 2609053890 | Ga0059124_1902 | | 410 | SP |  | | |
| PA-3 | *Escherichia coli* | 2 | 2608663970 | Ga0059088_11869 | | 427 | SP |  | | |
| NLAE-zl-G496 | *Shigella sonnei* | 2 | 2657180654 | Ga0104355_102164 | | 427 | SP |  | | |
| NLAE-zl-C269 | *Citrobacter* sp. | 927 | 2654374528 | Ga0105828_10254 | | 427 | SP |  | | |
|  |  |  |  |  | |  |  |  | | |
| **Euryarchaeota** |  |  |  |  | |  |  |  | | |
| Brm9 | *Methanobacterium formicicum* |  | 2631417653 | Ga0069308_111195 | |  |  |  | | |
| DSM 11855 | *Methanosarcina* sp. |  | 2596415729 | IE18DRAFT_00811 | |  |  |  | | |
|  |  |  | 2596417524 | IE18DRAFT_02606 | |  |  |  | | |
|  |  |  |  |  | |  |  |  | | |
| ^1^ This gene is not annotated as encoding a PME or having pfam01095 in IMG but was identified from the CAZy PLU database. The same gene is also found in the other *P. bryantii* strains. | | | | | | | | | | |
| ^2^ Other member of this clique (MB2005) lacks this gene and neighboring polygalactanase | | | | |  | | |  |  |  |
| ^3^ Three other members of this clique (AD3002, INlla16, WCD2001) lack this gene and neighboring polygalactanase | | | | | | | |  |  |  |
| ^4^ Not found in other rumen *L. mucosae* strains | |  |  |  | |  |  |  | | |
