# Supplementary material for: Occurrence and expression of genes encoding methyl-compound production in rumen bacteria
Source: Anim Microbiome. 2019 Nov 14;1:15. doi: 10.1186/s42523-019-0016-0 (PMC7807696; doi:10.1186/s42523-019-0016-0)
Supplement: Supplementary file 4 — Additional file 4: Table S3. Contigs and genome sizes of the three organisms identified from the MEC1 limited diversity metagenome. [file 42523_2019_16_MOESM4_ESM.docx]

| **Organism 1 GC~38%** | | **Organism 2 GC~52%** | | **Organism 3 GC~30%** | |  |
| --- | --- | --- | --- | --- | --- | --- |
| **Contig #** | **Size (bp)** | **Contig #** | **Size (bp)** | **Contig #** | **Size (bp)** |  |
| 1002 | 795500 | 1003 | 787651 | 1001 | 795605 |  |
| 1006* | 519031 | 1004 | 715695 | 1008 | 335751 |  |
| 1016 | 146765 | 1005 | 638227 | 1009 | 314420 |  |
| 1025 | 2299 | 1007 | 467607 | 1014 | 164243 |  |
|  |  | 1010 | 295033 | 1015 | 153656 |  |
|  |  | 1011 | 233662 | 1017 | 104643 |  |
|  |  | 1012 | 223529 | 1018 | 94589 |  |
|  |  | 1013 | 205007 | 1019 | 62781 |  |
|  |  | 1020 | 37735 | 1023 | 3086 |  |
|  |  | 1021 | 5653 | 1024 | 3045 |  |
|  |  | 1022* | 5022 | 1026* | 1490 |  |
|  |  |  |  |  |  |  |
| **Total** | 1463595 | **Total** | 3614821 | **Total** | 2033309 |  |

Supplementary Table 3. Contigs and genome sizes of the three organisms identified from the MEC1 limited diversity metagenome. Contigs containing the 16S rRNA gene sequence used for preliminary taxonomic identification are marked with an asterisk.
